# A nutritional biomarker score of the Mediterranean diet and incident type 2 diabetes: Integrated analysis of data from the MedLey randomised controlled trial and the EPIC-InterAct case-cohort study

**DOI:** 10.1371/journal.pmed.1004221

**Published:** 2023-04-27

**Authors:** Jakub G. Sobiecki, Fumiaki Imamura, Courtney R. Davis, Stephen J. Sharp, Albert Koulman, Jonathan M. Hodgson, Marcela Guevara, Matthias B. Schulze, Ju-Sheng Zheng, Claudia Agnoli, Catalina Bonet, Sandra M. Colorado-Yohar, Guy Fagherazzi, Paul W. Franks, Thomas E. Gundersen, Franziska Jannasch, Rudolf Kaaks, Verena Katzke, Esther Molina-Montes, Peter M. Nilsson, Domenico Palli, Salvatore Panico, Keren Papier, Olov Rolandsson, Carlotta Sacerdote, Anne Tjønneland, Tammy Y. N. Tong, Yvonne T. van der Schouw, John Danesh, Adam S. Butterworth, Elio Riboli, Karen J. Murphy, Nicholas J. Wareham, Nita G. Forouhi

**Affiliations:** 1 MRC Epidemiology Unit, University of Cambridge School of Clinical Medicine, Institute of Metabolic Science, Cambridge Biomedical Campus, Cambridge, United Kingdom; 2 Alliance for Research in Exercise, Nutrition and Activity, UniSA Clinical and Health Sciences, University of South Australia, Adelaide, Australia; 3 Nutritional Biomarker Laboratory, National Institute for Health Research Biomedical Research Centre, School of Clinical Medicine, University of Cambridge, Cambridge, United Kingdom; 4 Nutrition & Health Innovation Research Institute, School of Medical and Health Sciences, Edith Cowan University, Joondalup, Australia; 5 Medical School, University of Western Australia, Perth, Australia; 6 Navarra Public Health Institute, Pamplona, Spain; 7 CIBER of Epidemiology and Public Health (CIBERESP), Madrid, Spain; 8 Navarra Institute for Health Research (IdiSNA), Pamplona, Spain; 9 Department of Molecular Epidemiology, German Institute of Human Nutrition Potsdam-Rehbruecke, Nuthetal, Germany; 10 German Center for Diabetes Research (DZD), Munich-Neuherberg, Germany; 11 Institute of Nutritional Science, University of Potsdam, Nuthetal, Germany; 12 Key Laboratory of Growth Regulation and Translation Research of Zhejiang Province, School of Life Sciences, Westlake University, Hangzhou, China; 13 Epidemiology and Prevention Unit, Fondazione IRCCS Istituto Nazionale dei Tumori di Milano, Milano, Italy; 14 Unit of Nutrition and Cancer, Catalan Institute of Oncology—ICO, L’Hospitalet de Llobregat, Barcelona, Spain; 15 Nutrition and Cancer Group, Bellvitge Biomedical Research Institute—IDIBELL, L’Hospitalet de Llobregat, Barcelona, Spain; 16 Department of Epidemiology, Murcia Regional Health Council, IMIB-Arrixaca, Murcia, Spain; 17 Research Group on Demography and Health, National Faculty of Public Health, University of Antioquia, Medellín, Colombia; 18 Deep Digital Phenotyping Research Unit, Department of Precision Health, Luxembourg Insitute of Health, Strassen, Luxembourg; 19 Center of Epidemiology and Population Health UMR 1018, Inserm, Paris South—Paris Saclay University, Gustave Roussy Institute, Villejuif, France; 20 Department of Clinical Sciences, Lund University, Malmö, Sweden; 21 Vitas AS, Oslo, Norway; 22 Division of Cancer Epidemiology, German Cancer Research Center (DKFZ), Heidelberg, Germany; 23 Institute of Nutrition and Food Technology (INYTA) ‘José Mataix’, Biomedical Research Centre, University of Granada, Granada, Spain; 24 Instituto de Investigación Biosanitaria ibs.GRANADA, Granada, Spain; 25 Department of Nutrition and Food Science, University of Granada, Granada, Spain; 26 Cancer Risk Factors and Life-Style Epidemiology Unit, Institute for Cancer Research, Prevention and Clinical Network—ISPRO, Florence, Italy; 27 Department of Mental, Physical Health and Preventive Medicine, University “L. Vanvitelli”, Naples, Italy; 28 Cancer Epidemiology Unit, Nuffield Department of Population Health, University of Oxford, Oxford, United Kingdom; 29 Department of Public Health and Clinical Medicine, Family Medicine, Umeå University, Umeå, Sweden; 30 Unit of Cancer Epidemiology, Città della Salute e della Scienza University-Hospital, Turin, Italy; 31 Danish Cancer Society Research Center, Copenhagen, Denmark; 32 Department of Public Health, University of Copenhagen, Copenhagen, Denmark; 33 Julius Center for Health Sciences and Primary Care, University Medical Center Utrecht, Utrecht University, Utrecht, the Netherlands; 34 BHF Cardiovascular Epidemiology Unit, Department of Public Health and Primary Care, University of Cambridge, Cambridge, United Kingdom; 35 NIHR Blood and Transplant Research Unit in Donor Health and Genomics, Department of Public Health and Primary Care, University of Cambridge, Cambridge, United Kingdom; 36 British Heart Foundation Cambridge Centre of Excellence, Division of Cardiovascular Medicine, Addenbrooke’s Hospital, Cambridge, United Kingdom; 37 Department of Human Genetics, Wellcome Trust Sanger Institute, Hinxton, Cambridge, United Kingdom; 38 Health Data Research UK Cambridge, University of Cambridge, Cambridge, United Kingdom; 39 School of Public Health, Imperial College London, London, United Kingdom; Carolina Population Center, UNITED STATES

## Abstract

**Background:**

Self-reported adherence to the Mediterranean diet has been modestly inversely associated with incidence of type 2 diabetes (T2D) in cohort studies. There is uncertainty about the validity and magnitude of this association due to subjective reporting of diet. The association has not been evaluated using an objectively measured biomarker of the Mediterranean diet.

**Methods and findings:**

We derived a biomarker score based on 5 circulating carotenoids and 24 fatty acids that discriminated between the Mediterranean or habitual diet arms of a parallel design, 6-month partial-feeding randomised controlled trial (RCT) conducted between 2013 and 2014, the MedLey trial (128 participants out of 166 randomised). We applied this biomarker score in an observational study, the European Prospective Investigation into Cancer and Nutrition (EPIC)-InterAct case-cohort study, to assess the association of the score with T2D incidence over an average of 9.7 years of follow-up since the baseline (1991 to 1998). We included 22,202 participants, of whom 9,453 were T2D cases, with relevant biomarkers from an original case-cohort of 27,779 participants sampled from a cohort of 340,234 people. As a secondary measure of the Mediterranean diet, we used a score estimated from dietary-self report. Within the trial, the biomarker score discriminated well between the 2 arms; the cross-validated C-statistic was 0.88 (95% confidence interval (CI) 0.82 to 0.94). The score was inversely associated with incident T2D in EPIC-InterAct: the hazard ratio (HR) per standard deviation of the score was 0.71 (95% CI: 0.65 to 0.77) following adjustment for sociodemographic, lifestyle and medical factors, and adiposity. In comparison, the HR per standard deviation of the self-reported Mediterranean diet was 0.90 (95% CI: 0.86 to 0.95). Assuming the score was causally associated with T2D, higher adherence to the Mediterranean diet in Western European adults by 10 percentiles of the score was estimated to reduce the incidence of T2D by 11% (95% CI: 7% to 14%). The study limitations included potential measurement error in nutritional biomarkers, unclear specificity of the biomarker score to the Mediterranean diet, and possible residual confounding.

**Conclusions:**

These findings suggest that objectively assessed adherence to the Mediterranean diet is associated with lower risk of T2D and that even modestly higher adherence may have the potential to reduce the population burden of T2D meaningfully.

**Trial registration:**

Australian New Zealand Clinical Trials Registry (ANZCTR) ACTRN12613000602729 https://www.anzctr.org.au/Trial/Registration/TrialReview.aspx?id=363860.

## Abstract 2

Jakub G Sobiecki and team aim to develop a biomarker score able to discriminate between Mediterranean diet and habitual diet, and test its association with incident type 2 diabetes in a European cohort.

## Author summary

**Why was this study done?:**

- Epidemiological evidence has indicated that greater self-reported adherence to the Mediterranean diet may be associated with lower risk of new onset type 2 diabetes (T2D), but there has been uncertainty about the magnitude of association due to subjective reporting of diet.
- A combination of nutritional biomarkers could better assess diet–disease associations, but this approach has rarely been considered for overall diet quality, particularly for the association between the Mediterranean diet and T2D.

**What did the researchers do and find?:**

- Blood carotenoids and fatty acids can be used as an objective measure of adherence to the Mediterranean diet, as indicated by results from a trial (*n* = 128) of adopting Mediterranean diet with provision of its key foods to study participants. These biomarkers discriminated well between the trial participants under the Mediterranean diet intervention and those randomised to continuation of habitual diet (C-statistic = 0.88).
- In a study across 8 European countries (*n* = 22,202), adherence to the Mediterranean diet, estimated using a combination of nutritional biomarkers, was associated with lower risk of new onset T2D, with a stronger relationship compared to that with self-reported Mediterranean diet. The hazard ratios (HRs) (95% confidence interval (CI)) per standard deviation of adherence were 0.71 (0.65 to 0.77) and 0.90 (0.86 to 0.95), respectively.

**What do these findings mean?:**

- Adherence to the Mediterranean diet may be more beneficial for the primary prevention of T2D than previously estimated from observational dietary studies.
- Even small upward differences in objectively measured Mediterranean diet may be associated with a sizeable reduction of the risk of T2D at the population level.
- Causal interpretation of these findings is limited by potential measurement error in nutritional biomarkers, unclear specificity of the biomarker score to the Mediterranean diet, and possible residual confounding.

## Introduction

The Mediterranean diet is a dietary pattern typically characterised by high consumption of vegetables, legumes, fruit, nuts, grains, fish and seafood, virgin olive oil, and moderate intake of meat, dairy, and wine. It has been reported to be associated with decreased incidence of multiple noncommunicable diseases including type 2 diabetes (T2D) [1,2]. However, the evidence on the Mediterranean diet for prevention of T2D stems predominantly from observational research using self-reported dietary assessment, with modest effect sizes [3]. Dietary self-report is subject to systematic and random measurement error that may bias the associations with disease risk in an unknown direction [4]. Thus, the relationship between the Mediterranean diet and the incidence of T2D may have been misquantified. Uncertainty about the validity and size of this association limits robustness of the evidence for this dietary pattern and T2D incidence [3].

Nutritional biomarkers can complement the self-reported assessment of habitual dietary exposure. Dietary patterns, however, lack biologically plausible single biomarkers and, therefore, necessitate combining multiple analytes into composite biomarker scores [5,6]. Research on the derivation of such a score of the Mediterranean diet has had a number of limitations. Previous studies used mostly cross-sectional designs without external validation [7], and though evidence from feeding [5] or experimental [8] designs is promising, it has been hampered by challenges such as the different definitions of the Mediterranean diet or interventions used [5,9,10], the targeting of specific populations [5,8] and the use of subgroup analyses or not fully randomised enrolment of participants [5,11]. Overall, the validity and external generalisability of biomarkers of this dietary pattern reported in the literature remains largely unknown. Given these limitations, biomarker-based assessment of the Mediterranean diet has rarely been applied to the associations with disease outcomes [7] and never previously for T2D.

Our objectives were to derive a nutritional biomarker score that could discriminate between the Mediterranean diet intervention and the habitual diet arms of a randomised controlled trial (RCT), the MedLey trial, and to test the association of the biomarker score with incident T2D in a population-based observational study, the European Prospective Investigation into Cancer and Nutrition (EPIC)-InterAct case-cohort study. Additionally, we aimed to estimate the potential population impact of greater adherence to the Mediterranean diet, as assessed by the biomarker score, and future risk of T2D. We undertook a comparison between the biomarker score and a score based on self-report as previously reported in the EPIC-InterAct [12].

## Methods

### Study designs and populations

The overall study design and participant flows are displayed in S1 Fig. Briefly, we derived a nutritional biomarker score in the MedLey trial as an objective measure of adherence to the Mediterranean diet, and we applied the score in EPIC-InterAct to test its association with incident T2D. We developed 2 protocols for earlier iterations of this research idea as parts of broader statistical analysis plans. Key analytical decisions pertinent to the current analysis were prespecified in these documents (S1 Protocol).

The MedLey trial is an RCT that tested effects of the Mediterranean diet on cardiovascular risk factors [13] and cognitive function [14] among healthy adults aged ≥65 years from metropolitan Adelaide, Australia. We randomised 166 participants to either the Mediterranean diet with maintenance of baseline body weight or continuation of habitual diet for 6 months in 2013 and 2014 and measured circulating carotenoids [15–18] and fatty acids [15,19–22] to assess compliance [23,24]. Circulating carotenoids are biomarkers of dietary carotenoids or intake of fruits and vegetable [25,26]. Fatty acid profiles reflect across intakes of individual fatty acids and their metabolism, and are affected by dietary fat composition, intake of fish and shellfish, dairy products, nuts, and added fats, including olive oil [27–29]. The Mediterranean diet intervention consisted of fortnightly sessions with the study dietitian and provision of key shelf-stable foods amounting to 30% to 35% of estimated energy requirements: virgin olive oil, low-fat Greek yoghurt, unsalted nuts, canned legumes, and canned tuna. Participants in the habitual diet control group received gift vouchers to local food stores [23,24].

We used the MedLey trial to derive a biomarker score of discrimination between the Mediterranean and habitual diet arms based on end-of-study nutritional biomarkers. Among 137 trial completers out of 166 randomised individuals, we excluded participants with missing nutritional biomarker data (*n* = 4) and those with at least 1 biomarker value outside of the 25th percentile minus 3 times the interquartile range (IQR) or the 75th percentile plus 3 times the IQR (*n* = 5). The analytical sample (*n* = 128) included 67 participants in the Mediterranean diet group and 61 in the habitual diet arm (S1 Fig).

EPIC-InterAct Study is a case-cohort study of T2D nested within the EPIC study in 8 European countries (Denmark, France, Germany, Italy, the Netherlands, Spain, Sweden, and the United Kingdom) [30]. Baseline data collection took place during 1991 to 1998. We ascertained and verified 12,403 individuals with incident T2D from a cohort of 340,234 participants with stored blood samples in the EPIC study. Incident T2D cases were ascertained from a combination of sources, including self-report, primary and secondary care registers, drug registers, hospital admissions, and mortality data, as described in detail previously [30]. From the cohort of 340,234 participants, a centre-stratified subcohort was assembled by randomly selecting 16,835 individuals. A total of 16,154 participants remained in the subcohort after exclusions (*n* = 548 with prevalent diabetes; *n* = 133 with uncertain diabetes status). As a result, a case cohort was established (*n* = 27,779, including 12,403 incident T2D cases; S1 Fig) [30].

For the current analysis, we excluded participants with missing biomarkers required to calculate the biomarker score (*n* = 5,577), leaving 22,202 participants, with 9,453 participants who developed incident T2D and 13,313 subcohort participants (S1 Fig) followed over an average of 9.7 years. Excluded participants were similar to those included in the analytical sample in terms of the mean baseline age (53.0 versus 51.6 years), BMI (26.3 versus 26.1 kg/m^2^), and the proportion of women (59.0% versus 62.8%) based on subcohort distributions.

All participants provided written informed consent and studies were approved by local ethics committees. The MedLey trial was prospectively registered in the Australian New Zealand Clinical Trials Registry (ACTRN12613000602729). This study is reported as per the Strengthening the Reporting of Observational Studies in Epidemiology (STROBE) guideline (S1 Checklist) and Consolidated Standards of Reporting Trials (CONSORT) statement (S2 Checklist).

#### Nutritional and metabolic biomarkers

In the MedLey trial, venous blood samples were taken at baseline, 3 and 6 months post randomisation after 8 h of fasting, centrifuged and stored at −80°C. High-performance liquid chromatography (HPLC) with photo-diode array detection was used to assay carotenoids [31]. Individual fatty acids were assayed in erythrocyte membranes via direct transesterification as weight% of all the fatty acids measured, followed by gas chromatographic analysis at the Waite Lipid Analysis Service (Adelaide, Australia) [32]. Plasma total cholesterol (TC) was measured using a Siemens ADVIA chemistry analyser at SA Pathology (Adelaide, Australia). Laboratory staff were blinded to randomised intervention allocation.

In EPIC-InterAct, venous blood samples were collected at baseline. Plasma samples were stored in liquid nitrogen (up to −196°C). HPLC with ultraviolet light detection was used to measure carotenoids [33,34]. Individual plasma phospholipid fatty acids were assayed as mol% of all fatty acids using automated, high-throughput gas chromatography at the Medical Research Council Human Nutrition Research (Cambridge, United Kingdom) [35,36]. Serum TC was measured at the Stichting Ingenhousz Laboratory (Etten-Leur, the Netherlands) using Roche Hitachi Modular P. Haemoglobin A1c was measured in the erythrocyte fraction using the Tosoh-G8 analyser (Tosoh Bioscience, Japan). Laboratory staff were blinded to case status of participants, and samples were processed in a random order.

Five carotenoid variables were measured in both the MedLey trial and EPIC-InterAct: α-carotene, β-carotene, β-cryptoxanthin, lutein, and sum of lutein and zeaxanthin. For fatty acids measured in the MedLey trial, units were converted from weight% to mol% to harmonise the data with assays of fatty acids in the EPIC-InterAct. After exclusion of fatty acids with mean concentrations <0.05 mol% of total fatty acids measured, 24 fatty acids overlapped between the MedLey trial and EPIC-InterAct.

#### Measurement of covariates and self-reported diet

In the MedLey trial, questionnaires and physical examination were used to collect data on baseline characteristics. Self-reported diet was measured with 3-day weighed food diaries. Intakes of energy and ethanol were estimated using a local nutrient database [24].

In the EPIC study, questionnaires and physical examination were used at baseline to collect standardised information on covariates, including sociodemographic, medical and health behavioural factors, and anthropometry. Weight, height, and waist circumference were measured at baseline in all EPIC centres, except for Umea, Sweden, where waist circumference was not measured (*n* = 1,845) [30]. Subgroups of participants from France and the Oxford (UK) centre had self-reported anthropometry. Physical activity was assessed using a validated questionnaire [37]. Self-reported diet was measured using country-specific, validated food frequency questionnaires or diet histories. Estimation of intake of foods, energy, and nutrients was harmonised across the EPIC cohorts [38,39]. Information on family history of T2D was not collected in Italy, Spain, and Oxford and Heidelberg centres. Information on current use of vitamin or mineral supplements was not collected in Heidelberg. Covariates had <30% missing data within countries except for Germany with 45% of missing data for vitamin/mineral supplement use and family history of T2D. The prevalence of missing information for all countries combined was the highest for family history of T2D (22%), and otherwise up to 9% (dietary supplements), with several covariates having complete data for all participants.

As a measure of self-reported adherence to the Mediterranean diet, we used a score that EPIC-InterAct previously derived (S1 Text) and reported an association with lower T2D incidence [12].

### Statistical analysis

Stata 16.1 and R 4.0.2 were used for statistical analysis.

#### Derivation of the biomarker score

We imputed values of fatty acids below the limit of detection using quantile regression imputation [40]. In the MedLey trial, values were imputed for 5 out of the 24 fatty acids and the proportion of missingness was the highest for C17:1 at 21% and otherwise <13%. In EPIC-InterAct, values were imputed for 16 fatty acids, and the corresponding proportions of missingness were 12% (also for C17:1) and <6%, respectively. Fatty acids were re-scaled to sum up to 100% within the sets of fatty acids overlapping between the MedLey trial and EPIC-InterAct. Concentrations of circulating carotenoids (ng/mL) were adjusted for TC using the residual method in order to account for their correlations with dietary carotenoids [41].

We used logistic elastic net regression to derive the biomarker score in MedLey trial [42,43]. Natural-log-transformed nutritional biomarkers measured at 6 months were used to predict the binary randomised assignment to the Mediterranean or habitual diet groups. We repeated the elastic net regression with random cross-validation 1,000 times to stabilise the variable selection and included in the final model the predictors with selection rate ≥90% [43]. Ridge regression was used to calculate penalised coefficients of the final model [44]. We selected the predictors from 5 carotenoid and 24 fatty acid variables and 406 pairwise interaction terms between them. The biomarker score was calculated as a linear prediction from the ridge regression model. Discriminatory performance was assessed by calculating 5-fold cross-validated C-statistic. The associated 95% confidence interval (CI) was estimated based on the Hanley and McNeil variance formula [45]. Additional details on derivation of the biomarker score are provided in the Supporting information (S1 Text).

#### Associations of the biomarker score with incident T2D

In EPIC-InterAct, individual nutritional biomarkers were Winsorised at 4 standard deviations (SDs) below or above the subcohort means and were then used to calculate the biomarker score with the scoring algorithm developed as described above. The biomarker score was standardised using the means and SD and categorised into quintiles based on the distribution in the subcohort. We performed Prentice-weighted Cox regression analysis with a robust variance estimator to estimate hazard ratios (HRs) for the association between the biomarker score and incident T2D [46]. Country-specific HRs were estimated and pooled using random-effects meta-analysis, followed by calculation of the 95% confidence and prediction intervals [47]. Restricted cubic splines with 5 knots were used to assess potential nonlinearity of the association between the standardised biomarker score and T2D incidence. Country-specific estimates of the spline parameters were pooled with multivariate random-effects meta-analysis [48].

The multivariable-adjusted model included the following covariates: age (as the underlying timescale), sex, recruitment centre, prevalent comorbidity (cancer, cardiovascular disease, hypertension, and hyperlipidaemia), family history of T2D, smoking status (never, former, and current smoker), physical activity index (inactive, moderately inactive, moderately active, and active), seasonality (sine and cosine function of the day of the year of blood draw), fasting status (<3, 3–6, >6 h), current use of vitamin or mineral supplements, marital status (single, married or cohabiting, divorced or separated, and widowed), educational attainment (none, primary school, technical or professional school, secondary school, and post-secondary school education), current employment, and in women, menopausal status (pre-, peri-, postmenopausal, and bilateral oophorectomy) and current hormone replacement therapy use. A further model was fitted with adjustment for adiposity (BMI and waist circumference as continuous variables; main analytical model). Additional models explored the effects of adjustment for biomarkers constituting the biomarker score. Continuous covariates were Winsorised at 4 SDs below or above the subcohort means. For comparison with the biomarker-based assessment, we estimated the HR for the association with incident T2D of the standardised score of self-reported Mediterranean diet using the main analytical model.

We examined multiplicative interactions of the biomarker score with the following covariates: age, sex, BMI, seasonality, fasting status, use of dietary supplements, physical activity, and smoking status using the adiposity-adjusted model (excluding waist circumference for interaction by BMI) and variable specifications as outlined above. Missing covariate data were imputed by multiple imputation using chained equations in 10 datasets [49], accounting for exposures (biomarker score, nutritional biomarkers), the outcome, model covariates, interaction terms, and country-specific case-cohort design [50]. We also performed a complete-case analysis.

To assess the potential public health impact of a feasible shift in the average population adherence to the Mediterranean diet, we modelled the population attributable fraction (PAF) [51] if the value of the biomarker score for each individual in the study population were to increase by 10 percentiles, assuming a causal relationship. The PAF was estimated in the EPIC-InterAct subcohort separately in each country in the adiposity-adjusted multivariable model, and then combined across countries using random-effects meta-analysis [47]. We used country-specific inverse-probability weights to account for the case-cohort design [52,53]. To facilitate interpretation of the PAF by comparison with an established risk factor, we estimated this measure for a 10 percentile lower BMI after removing from the multivariable model the biomarker score, waist circumference, and physical activity.

#### Sensitivity analyses

We performed several sensitivity analyses to assess the robustness of the main findings. We repeated the derivation and assessment of discriminatory performance of the biomarker score in the MedLey trial with several alternative analytical decisions in the elastic net regression (S1 Text), followed by longitudinal analysis in EPIC-InterAct for each alternative score. Using the primary biomarker score in EPIC-InterAct, time-varying effects were assessed by splitting follow-up time at 7 years and performing stratified analysis. Potential reverse causation bias was evaluated by separately (i) excluding the first 2 years of follow-up; (ii) excluding participants with prevalent major disease conditions (cancer, myocardial infarction, or stroke); and (iii) excluding participants with baseline haemoglobin A1c concentrations ≥ 6.5% (48 mmol/mol).

#### Analyses conducted in response to peer review comments

We performed a graphical assessment of calibration of the biomarker score model for prediction of randomised assignment in the MedLey trial [54,55]. We tested the discriminatory performance of the biomarker score between ≥90th percentile versus ≤10th percentile of self-reported adherence to the Mediterranean diet (S1 Text). We assessed the influence of use of pharmacological treatments in our analyses by evaluating: (i) distributions of users of medications by MedLey trial arms (chi-squared test); (ii) multiplicative interactions between use of medications and the biomarker score in logistic regression models for prediction of randomised assignment; (iii) re-derivation of the biomarker score with adjustment of the coefficients for use of medications in the MedLey trial and its impact on the association between the score and incidence of T2D in EPIC-InterAct; and (iv) additional adjustments of the main analytical model in EPIC-InterAct for use of medications in subsets of participants with information available on these covariates.

### Patient and public involvement

The MedLey trial intervention was piloted in a group of volunteers that led to minor adjustments in its design and delivery [56]. Patients or public were otherwise not involved in study design.

## Results

Compared to the MedLey trial participants, the EPIC-InterAct subcohort members were younger (mean age 52 years, EPIC-InterAct, versus 71 years, MedLey trial), had lower tertiary educational attainment (20% versus 53%), were less likely to have a family history of T2D (18% versus 30%), and had higher prevalence of hypertension (36% versus 19%) and hyperlipidaemia (38% versus 17%). The study populations were similar with regards to dietary and cardiometabolic phenotypes (**Table 1**). The Mediterranean diet intervention in the MedLey trial resulted in increases in the concentrations of ß-carotene, lycopene, C22:6-n3 (docosahexaenoic), C20:2-n6 (eicosadienoic), and long- and very-long chain monounsaturated fatty acids, and decreases in the concentrations of C22:5-n6 (osbond), C17:1 (heptadecenoic) and several saturated fatty acids (S2 Fig). The effect sizes relative to the habitual diet control group ranged from −0.67 SD for C14:0 (myristic) to 0.76 SD for C20:1 (gondoic acid). Blood levels of these biomarkers varied between the MedLey trial and EPIC-InterAct. For example, comparing median concentrations in the MedLey trial habitual diet group and ranges of medians by country in the EPIC-InterAct subcohort, we observed differences for ß-carotene (726 and 144 to 419 ng/mL), lycopene (110 and 172 to 348 ng/mL), C24:1 (1.22 and 0.32 to 0.37%mol), and C24:0 (1.07 and 0.20 to 0.26%mol) fatty acids (S1 Table).

**Table 1:** Baseline characteristics of participants of the MedLey randomised partial-feeding controlled trial of the Mediterranean diet and the EPIC-InterAct case-cohort study*.

	MedLey trial	EPIC-InterAct	
		Subcohort	Cases of T2D
Number of participants	128	13,313	9,453
Age, years	71 (5)	52 (9)	55 (8)
Women, %	54	63	50
Postmenopausal, %^†^	100	44	60
Hormone therapy use, %^†^	5	14	14
Current smokers, %	0	26	28
Moderately active or active, %	-^‡^	43	38
Tertiary education, %	56	20	13
Currently employed, %	21	67	58
Family history of T2D, %	31	18	34
Disease history, %			
Hypertension	36	19	39
Hyperlipidaemia	38	17	27
Cardiovascular disease	5.0	1.9	4.0
Cancer	-	2.4	2.5
Mediterranean diet score, 0–18 points	9.7 (2.6)	8.8 (3.1)	8.5 (3.2)
Score components, g/1,000 kcal			
Vegetable	83 (67)	94 (64)	92 (66)
Legumes	8.2 (17.4)	9.2 (13.2)	9.4 (14.3)
Fruits and nuts	136 (75)	123 (99)	119 (102)
Grains and grain products	72 (39)	104 (41)	103 (43)
Fish and shellfish	24 (26)	18 (16)	20 (18)
Meat and meat products	35 (32)	52 (24)	58 (25)
Milk and milk products	130 (95)	163 (114)	160 (122)
Olive oil	2.0 (3.4)	4.3 (6.3)	4.0 (6.3)
Ethanol	4.6 (5.0)	6.0 (7.7)	6.5 (8.7)
Dietary supplement use, %	66	39	41
Body mass index, kg/m^2^	26.5 (3.5)	26.1 (4.2)	29.9 (4.7)
Waist circumference, cm	90 (13)	87 (13)	98 (12)
Haemoglobin A1c, mmol/mol	-	35.9 (4.9)	43.1 (11.1)
≥6.5% (48 mmol/mol), %	-	1	17
Total cholesterol, mmol/L	5.2 (0.9)	5.9 (1.1)	6.1 (1.2)
HDL cholesterol, mmol/L	1.65 (0.44)	1.50 (0.42)	1.25 (0.37)
Triglycerides, mmol/L	1.13 (0.45)	1.33 (0.91)	1.98 (1.41)

*Values are means (standard deviations) or percentages. The EPIC-InterAct subcohort participants and incident cases of T2D were independently sampled from the underlying EPIC cohort. As a feature of the case-cohort design, 564 incident cases included in the analysis were simultaneously subcohort participants.

^†^Calculated in women.

^‡^Data unavailable in the MedLey trial.

EPIC, European Prospective Investigation into Cancer and Nutrition; HDL, high density lipoprotein; T2D, type 2 diabetes.

### Derivation of the biomarker score

**Fig 1:**
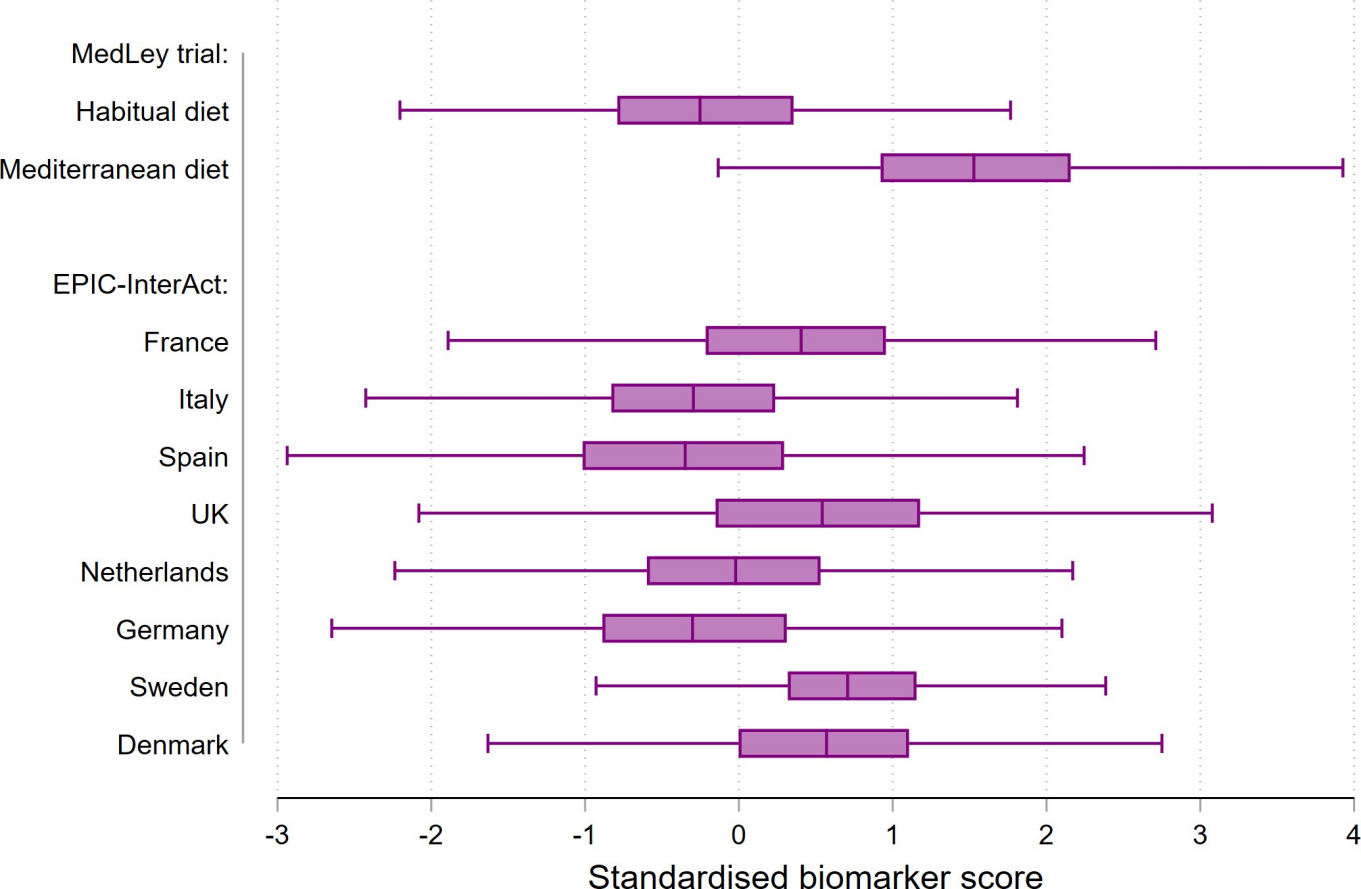
Nutritional biomarker score of the Mediterranean diet: study-specific distribution by the MedLey trial arms post-intervention and the EPIC-InterAct subcohort by country at baseline. The biomarker score was derived as a discriminatory model between the Mediterranean and habitual diet in the MedLey randomised partial-feeding controlled trial. Circulating carotenoids and fatty acids were used to calculate the score as linear predictions from the discriminatory model. The score was standardised separately within the MedLey trial and the EPIC-InterAct subcohort. Boxes denote the IQR and medians inside; and whiskers, values up to 1.5 IQR outside of these percentiles. EPIC, European Prospective Investigation into Cancer and Nutrition; IQR, interquartile range; UK, United Kingdom.

The biomarker score consisted of a linear combination of 23 biomarkers in total (S2 Table). The score distinguished 2 arms of the Mediterranean and habitual diet groups with C-statistic = 0.88 (95% CI: 0.82 to 0.94) and moderate calibration (S3 Fig). We did not find any evidence of interaction between medication use and the biomarker score (S3 Table). After standardisation of the score in the MedLey trial, mean end-of-trial score values were higher by 1.81 (95% CI: 1.45 to 2.18) points in the Mediterranean than the habitual diet group. In EPIC-InterAct, baseline medians of the biomarker score standardised using the overall subcohort distribution ranged between countries from −0.35 in Spain to 0.71 SD in Sweden (**Fig 1** and S4 Table). The C-statistic values for discrimination between extreme categories of self-reported Mediterranean diet by the biomarker score were 0.63 (95% CI: 0.38 to 0.87; *n* = 42) in the MedLey trial baseline sample and 0.59 (95% CI: 0.58 to 0.61; *n* = 4,298) in the EPIC-InterAct subcohort, with country-specific values ranging from 0.54 in Germany to 0.68 in Spain.

### The association of the biomarker score with incident T2D

**Fig 2:**
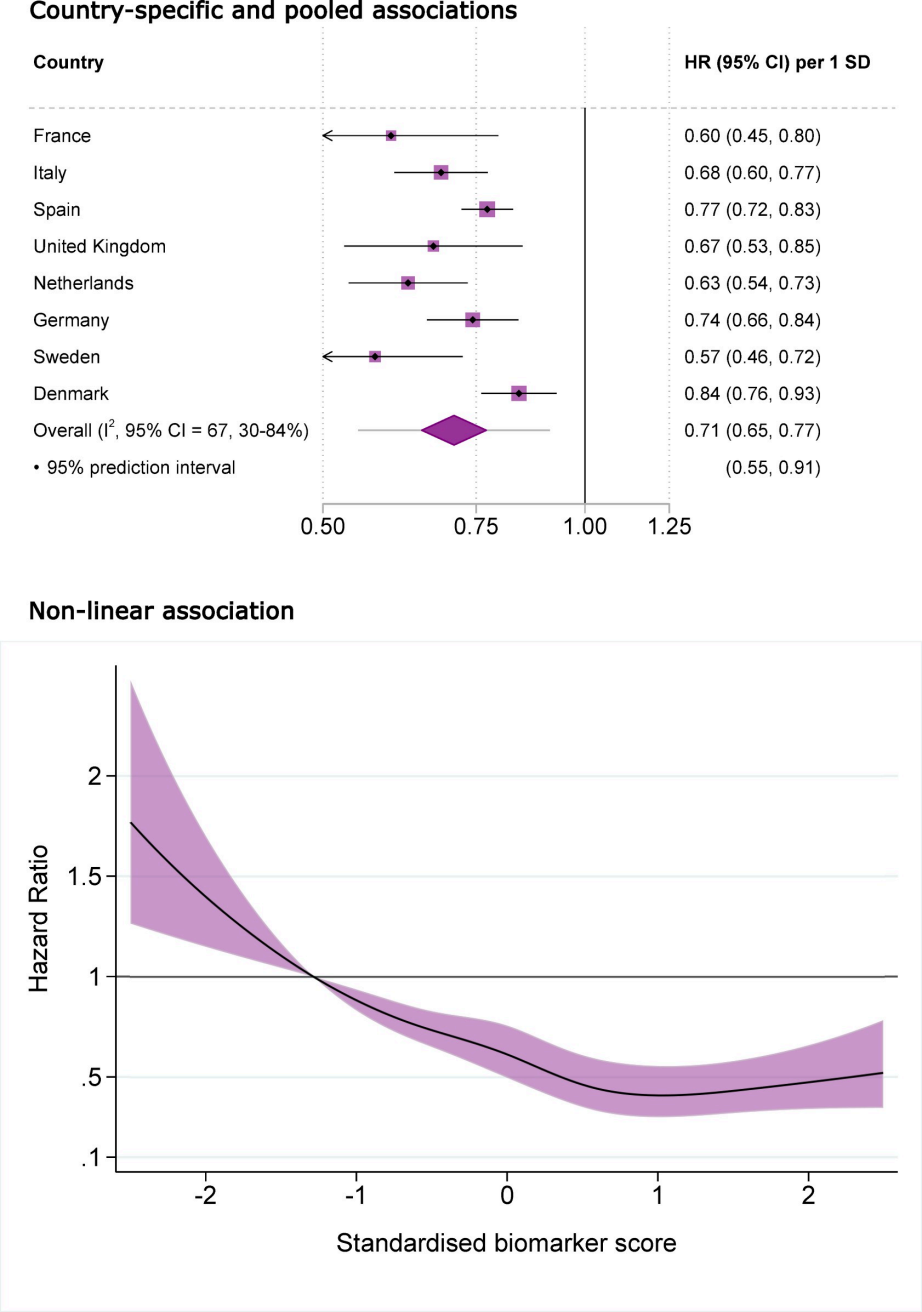
Association between the nutritional biomarker score of the Mediterranean diet and incidence of T2D in the EPIC-InterAct case-cohort study (*n* = 22,202). The biomarker score was derived as a discriminatory model between the Mediterranean and habitual diet in the MedLey randomised partial-feeding controlled trial. Circulating carotenoids and fatty acids were used to calculate the score as linear predictions from the discriminatory model. Associations were assessed with the Prentice-weighted Cox regression and pooled by random-effects meta-analysis. Top: The diamond and error bars of the pooled estimate represent the 95% confidence and prediction intervals. Bottom: Restricted cubic splines with 5 knots were used to model the nonlinear association. The *p*-value for the test of nonlinearity was 0.044. Black solid line represents point estimates of HRs and purple area denotes the 95% CI. The 10th percentile of the subcohort distribution was used as reference. Associations were adjusted for: age (as timescale), sex, recruitment centre, prevalent cancer, cardiovascular disease, hypertension and hyperlipidaemia, familial history of type 2 diabetes, smoking status (never, former, current smoker), physical activity index (inactive, moderately inactive, moderately active, active), seasonality (sine and cosine function of the day of the year), fasting status (<3, 3–6, >6 h), current use of vitamin or mineral supplements, marital status (single, married or cohabiting, divorced or separated, widowed), educational attainment (none, primary school, technical or professional school, secondary school, post-secondary school education), current employment, body mass index and waist circumference, and in women, menopausal status (pre-, peri-, postmenopausal, bilateral oophorectomy) and current hormone replacement therapy use. CI, confidence interval; HR, hazard ratio; SD, standard deviation; T2D, type 2 diabetes.

The biomarker score of the Mediterranean diet was inversely associated with incident T2D (**Table 2**). In the adiposity-adjusted multivariable model, the HR (95% CI) for the top fifth of the biomarker score compared to the bottom fifth was 0.38 (0.30 to 0.50) (p trend = 0.012). The HR (95% CI) per 1 SD was 0.71 (0.65 to 0.77) with inverse associations in all countries, moderate heterogeneity between country-specific estimates (I^2^ = 67%) and a 95% prediction interval of 0.55 to 0.91 (**Fig 2**). We found weak evidence of departure from linearity (p nonlinearity = 0.044, **Fig 2**) where the inverse association levelled off in approximately the upper fifth of the distributions of the score. The association remained statistically significant after additional adjustments for individual and all biomarkers or interaction terms included in the score (S5 Table). The estimated PAF (95% CI) was 11% (7% to 14%), indicating that the incidence of T2D could be reduced by 11% if the biomarker score were increased by 10 percentiles, assuming a causal relationship. For comparison, the estimated PAF (95% CI) for a 10 percentile lower BMI was 28% (20% to 35%).

**Table 2:** Associations between the nutritional biomarker score of the Mediterranean diet* with incidence of T2D in the European Prospective Investigation into Cancer and Nutrition-InterAct (*n* = 22,202).

Model	Quintiles					p_trend_^‡^	Per 1 SD	I^2^, % (95% CI)
	Q1	Q2	Q3	Q4	Q5			
Number of cases	2,779	1,954	1,698	1,508	1,514			
IR per 100,000 person-years	572	407	286	252	268			
Pooled HRs (95% CIs)^†^								
Age, sex, and centre adjusted	1.0 (Ref.)	0.64 (0.58–0.69)	0.49 (0.44–0.54)	0.40 (0.34–0.47)	0.34 (0.28–0.41)	<0.001	0.65 (0.61–0.69)	69 (39–85)
Multivariable adjusted^†^	1.0 (Ref.)	0.66 (0.60–0.73)	0.53 (0.46–0.60)	0.43 (0.37–0.49)	0.36 (0.29–0.45)	<0.001	0.67 (0.62–0.72)	67 (33–84)
+adiposity	1.0 (Ref.)	0.69 (0.62–0.77)	0.59 (0.52–0.67)	0.48 (0.40–0.58)	0.38 (0.30–0.50)	0.012	0.71 (0.65–0.77)	67 (30–84)

*The biomarker score was derived as a discriminatory model between the Mediterranean and habitual diet in the MedLey randomised partial-feeding controlled trial. Circulating carotenoids and fatty acids were used to calculate the score as linear predictions from the discriminatory model.

^†^Hazard ratios were pooled from country-specific estimates. Multivariable adjusted model included the following covariates: age (as timescale), sex, recruitment centre, prevalent cancer, cardiovascular disease, hypertension and hyperlipidaemia, familial history of T2D, smoking status (never, former, current smoker), physical activity index (inactive, moderately inactive, moderately active, active), seasonality (sine and cosine function of the day of the year), fasting status (<3, 3–6, >6 h), current use of vitamin or mineral supplements, marital status (single, married or cohabiting, divorced or separated, widowed), educational attainment (none, primary school, technical or professional school, secondary school, post-secondary school education), current employment, and in women, menopausal status (pre-, peri-, postmenopausal, bilateral oophorectomy), current hormone replacement therapy use. Adjustment for adiposity included body mass index and waist circumference.

^‡^ Generalised least-squares trend estimation method was used to calculate *p*-values for a linear trend over an ordinal variable of median biomarker scores of the 5 quintile groups.

CI, confidence interval; HR, hazard ratio; IR, incidence rate; SD, standard deviation; T2D, type 2 diabetes.

The main result was robust to multiple sensitivity analyses which explored the effects of time of follow-up, reverse causation, alternative analytical decisions at the stage of derivation of the biomarker score, and additional adjustment for use of medications (S5 Table). Among the potential effect modifiers, we found evidence of interaction of the biomarker score with age and the use of dietary supplements (S6 Table). The HR (95% CI) per 1 SD of the biomarker score was 0.76 (0.69 to 0.84) in supplement users and 0.69 (0.62 to 0.75) in non-users. The stratum-specific estimates by age at baseline <45, 45–60, and >60 years were 0.54 (0.42 to 0.69), 0.74 (0.69 to 0.80), and 0.74 (0.67 to 0.82), respectively. The results from complete-case analysis were similar to the multiple imputation estimates (S6 Table). The HR (95% CI) per 1 SD of the score of self-reported Mediterranean diet was 0.90 (0.86 to 0.95).

## Discussion

In the current research, we combined information from experimental and observational studies to investigate the association between a composite biomarker score of adherence to the Mediterranean diet and incident T2D. The key findings were that a biomarker score derived within the MedLey RCT had a high discriminatory performance between the Mediterranean and habitual diet arms, and that when this biomarker score was applied to the pan-European EPIC-InterAct Study, there was an inverse association with incident T2D. The 95% prediction interval for this association did not include the null, suggesting that our finding would be expected to be replicated in similar populations [57]. The strength of the association was greater in magnitude than that between the self-reported Mediterranean diet and T2D. Moreover, higher adherence to the Mediterranean diet (as reflected by a 10-percentile higher value of the biomarker score) could reduce the incidence of T2D by 11%, assuming a causal relationship which cannot be confirmed by the present study.

### Comparison with previous studies

Attempts to derive biomarkers of the Mediterranean diet and other dietary patterns have previously been largely confined to metabolomic profiling using cross-sectional designs [7,58]. In a subgroup analysis in one of the centres of the PREDIMED trial, urinary metabolomic profiles at 1 or 3 years post-randomisation were able to correctly classify 93%, 85%, and 68% of participants to their respective intervention arms of the Mediterranean diet with either olive oil or nuts and the control lower fat diet [8]. Only 2 previous studies considered scores comprised of nutritional biomarkers [5,59], one of which used a hypothesis-free approach that was likely prone to confounding by non-dietary regulation of nutritional biomarkers [59]. The second study used a novel feeding design of habitual diets and performed data-driven variable selection from a wider range of nutritional biomarkers than available in our investigation: serum phospholipid fatty acids, carotenoids, tocopherols, retinol, B vitamins, and 24-h urinary nitrogen, sodium, potassium, and energy expenditure estimated from the doubly labelled water technique [5]. The study derived a biomarker score that was well correlated with quantified habitual adherence to the Mediterranean diet (r_cross-validated_ = 0.60). It included 2 circulating carotenoids and 7 fatty acids that constituted 81% of the prediction performance, as well as urinary potassium and energy expenditure [5]. This finding corroborates our results and the hypothesis that combinations of nutritional biomarkers, and in particular circulating carotenoids and fatty acids, can be used to objectively assess adherence to the Mediterranean diet [15–22]. Importantly, it also highlights the remaining biomarkers not selected into the score as potentially having limited value for assessment of the Mediterranean dietary pattern, which is supported by interventional evidence for some of these analytes, e.g., for tocopherols [16]. Studies on other dietary patterns likewise suggest utility of combinations of carotenoids and fatty acids for their objective assessment [5,60–62]. It is prudent for future research on biomarker scores of the Mediterranean diet to evaluate further groups of nutritional biomarkers in a data-driven manner. For example, biomarkers of polyphenols are promising [21,63], however, the evidence is limited and their utility has so far not been assessed in the context of multivariate modelling.

Our work on derivation of the nutritional biomarker score of the Mediterranean diet adds to the previous literature by incorporating the strength of an RCT. Owing to the RCT design of the MedLey trial, we were able to minimise confounding by non-nutritional factors, and thus derive a biomarker score with| potentially improved generalisability to external populations. A further novelty of our work is the application of the experimentally derived score to prospective associations between dietary patterns at large and disease outcomes. To our knowledge, the only other example of derivation and application of a putative biomarker of the Mediterranean diet to associations with disease endpoints was a metabolomics-based analysis in the Spanish PREDIMED study and prospective cohorts in the USA. Similar to our analysis, it observed inverse associations for cardiovascular disease using the metabolite score observationally derived in PREDIMED across all cohorts (HR range per 1 SD: 0.71 to 0.86) and weaker or null associations when using the self-reported Mediterranean diet [64]. Of note, these results also provide evidence in support of transferability of objective exposure assessment measures across populations for epidemiological investigations. This is particularly pertinent to the current manuscript in which we were unable to test the association of the biomarker score with incidence of T2D in the target population of the MedLey trial.

For incident T2D, the PREDIMED trial reported a 30% risk reduction in the Mediterranean diet intervention arms relative to the control lower fat diet group (273 incident cases in 3,541 participants) [65]. The CORDIOPREV trial reported a statistically nonsignificantly higher incidence of T2D in the Mediterranean diet arm compared to the lower fat control group (HR 1.35; 95% CI: 0.91 to 2.01; 107 cases in 462 participants) [66]. This finding from a secondary CVD prevention trial among participants predominantly with pre-diabetes (85% prevalence) is of lower relevance to prevention of T2D in the general population than that of the PREDIMED trial. Beyond moderating effects of population characteristics, the result of the CORDIOPREV trial may have been driven towards favouring the lower fat diet by a small mean weight loss over 5 years (−1.14 kg) compared to a small average weight gain in the Mediterranean diet arm (+0.78 kg) [66]. Inverse associations between the self-reported Mediterranean diet and incident T2D in middle-aged adults have previously been reported in EPIC-InterAct [12] and other prospective cohorts [2]. The largest reduction in the incidence between extreme categories of the self-reported adherence to the Mediterranean diet was 25% (top versus bottom fifth) [67], which is a substantially smaller effect size than the 62% observed in the current study using the biomarker score.

### Strengths and limitations

The major strength of the current research was the use of a novel analytical approach that combined the derivation of an objective measure of the Mediterranean diet in a partial-feeding study (the MedLey RCT) and its application in a large observational study (the EPIC-InterAct Study). The RCT compared the effects of this dietary pattern (without weight loss) with continuation of habitual diet on nutritional biomarkers. Such an experimental design allowed us to derive a biomarker score of the Mediterranean diet in a manner that was free from influences of other dietary and non-dietary factors, while using a control group suitable for application of the score to study participants in an observational setting in Western countries. We used a set of biomarkers that reflect dietary exposures over the past weeks or months [68] that is a desirable timeframe for assessment of habitual diet for epidemiological applications. Our analysis was based on the largest study to date of nutritional biomarkers and T2D, including over 9,000 incident cases. Among other strengths, our observational analyses adjusted for a comprehensive range of potential confounding factors and included several sensitivity analyses, compared the main results against the Mediterranean diet assessed by self-report, and modelled the population impact of greater adherence to the Mediterranean diet on future T2D risk.

Our research had several limitations. The intervention in the MedLey trial was administered at one level of intensity. This allowed for modelling of the Mediterranean diet only as a binary variable and precluded objective evaluation of the dose-response relationship between the adherence to this dietary pattern and the biomarker score [69]. Second, the MedLey trial was a partial-feeding RCT that may have resulted in lower adherence to the dietary components not provided (e.g., fruits and vegetables) than the food items provided as part of the Mediterranean diet intervention. Third, we used a combination of candidate biomarkers of intake, concentration, and function that are not only affected by dietary intakes, but also bioavailability, endogenous synthesis, genetic variation, homeostatic control, and nutrient metabolism [27,70,71]. Thus, changes in nutritional biomarkers in the MedLey trial may have represented a metabolic response to a healthy diet, rather than a specific biomarker profile indicative of adherence to the Mediterranean diet. Fourth, we were unable to assess whether participants in the intervention group reached equilibrium in concentrations of nutritional biomarkers and values of the biomarker score. Fifth, the trial experienced a moderate degree of drop-outs between the randomisation and the end-of-trial assessment (20% in each arm, including missing biomarker data). Both the unknown equilibrium status and the loss to follow-up [72] may have biased the derivation of the RCT score and evaluation of its performance as a classifier. The biomarker score had limited utility in discriminating between extreme categories of self-reported adherence to the Mediterranean diet; however, the measurement error due to subjective reporting and potentially insufficient construct validity of the self-reported Mediterranean diet score may have meant the C-statistics were underestimated. Sensitivity and specificity of the biomarker score, as well as external validity, remain unknown at present and require evaluation of the score in external trials of interventions with the Mediterranean diet and other dietary patterns. We also note that the MedLey trial used canned fish and legumes as a pragmatic, nontraditional approach to implementation of these components of the Mediterranean diet; however, we would not expect this method of preservation to have a materially differential effect on the biomarkers used compared to cooked fresh fish and beans cooked from dry.

Limitations in the observational research in EPIC-InterAct included residual confounding as a potential source of bias with an overall unclear direction and magnitude. It was unlikely to fully account for the inverse association given its large effect size and the effect sizes of risk factors for T2D previously reported in the literature [73,74]. However, the above mentioned potentially limited specificity may have contributed to positive residual confounding. There may have been degradation of the nutritional biomarkers despite the storage in liquid nitrogen, but we would not expect any bias in the relative risk estimate assuming the degradation rates were non-differential by the incident case status or biomarker level. The random measurement error in the nutritional biomarkers would be expected to bias the association towards the null when considered in isolation from other sources of error, but the context of multivariable statistical modelling precludes any inference on the direction of the potential bias [4]. The differential misclassification in the ascertainment of the outcome also may have biased the results in an unknown direction. We can speculate that higher adherence to the Mediterranean diet may have been associated with greater health-consciousness and healthcare-seeking behaviours, higher likelihood of T2D diagnosis, and an underestimation of the inverse association. We standardised the measures of the Mediterranean diet to improve interpretability of the results for the biomarker score over its original scale and to allow for a comparison of the effect size with that of the self-reported Mediterranean diet. This approach conditioned the effect sizes on the underlying distributions of the exposure variables in EPIC-InterAct which may have limited our quantitative and comparative interpretation [75]. Similarly, our modelling of the PAF was limited by using a relative scale of the biomarker score to quantify its potential impact on population-level prevention of T2D. Public health relevance of this result was further constrained by the fact that the current evidence-base does not enable a direct interpretation of how to achieve the 10-percentile change in the score. Our analysis was not adjusted for use of medications due to incomplete availability of data on these covariates across recruitment centres (<30% participants). However, sensitivity analyses adjusted for use of medications in subsets of study participants showed no appreciable differences compared with the primary results, reducing the potential for residual confounding by medication use. The use of dietary supplements was available as a binary variable that allowed only for a crude assessment of effect modification. Finally, reliability of the externally derived biomarker score applied to biomarker assays used in EPIC-InterAct remains unknown.

### Implications of this research

The analysis of dietary patterns aims to evaluate the cumulative impact of dietary exposures on disease risk to inform development of dietary guidelines [1]. High-quality evidence on dietary patterns and the primary prevention of T2D is lacking, partly because of limitations of dietary assessment methods [3]. Self-reported tools have been used almost exclusively in the published studies on this topic [3,76]. We have developed a method of objective assessment of the Mediterranean diet via nutritional biomarkers in order to investigate the association of this dietary pattern and T2D. Our approach of using objective biomarkers yielded substantially greater magnitude and stronger inverse associations than for the Mediterranean diet assessed with subjective methods, also consequently addressing the limitation of small effect sizes often observed in nutritional epidemiology [77]. Our modelling indicates that a modest, 10-percentile increase in the objectively assessed Mediterranean diet could potentially avert 11% of new T2D cases. In comparative analysis, this effect size was approximately 40% of the estimated effect of decreasing mean BMI by 10 percentiles as an example of a well-established, causal risk factor for T2D with a large effect size [74]. Of note, the inverse associations of the biomarker score with T2D in the current study were independent from measured adiposity, with no evidence of effect modification by BMI. Therefore, our finding suggests that a sizeable decrease in the incidence of T2D could potentially be achieved through improved adherence to the Mediterranean diet even in the absence of modification of body weight and across the spectrum of adiposity in the population.

Our integrative approach of using a trial and an observational study with nutritional biomarkers strengthens the evidence for the utility of biomarkers in research on dietary patterns. Further investigations are warranted for a broader use of biomarkers to monitor dietary adherence in research, clinical settings, and potentially of preventive interventions in the real world. Our primary finding was consistent with interventional evidence from a Mediterranean (Spanish) population [65], and it lends itself to consideration for developing dietary guidelines, public health policy, and personalised dietary advice.

## Conclusions

The findings of the current study have demonstrated the utility of combining circulating carotenoids and fatty acids as a composite biomarker of the Mediterranean diet. The inverse association between a biomarker score of this dietary pattern and T2D was approximately 3-fold larger than for adherence to the Mediterranean diet estimated from dietary self-report, thus raising the possibility that adherence to the Mediterranean diet is likely to be more beneficial for the primary prevention of T2D than previously estimated from observational dietary studies. These results add to the evidence in favour of adopting a Mediterranean-type diet in Western European adults for the prevention of T2D.

## Supporting information

S1 Checklist STROBE Statement—Checklist of items that should be included in reports of cohort studies. (DOCX)

S2 Checklist CONSORT 2010 checklist of information to include when reporting a randomised trial. (DOCX)

S1 Text Supplementary methods. (DOCX)

S1 Table Medians (25th, 75th percentile) of circulating carotenoids and fatty acids: the MedLey trial post-intervention and the EPIC-InterAct subcohort at baseline. Abbreviations: Hab, habitual; Med, Mediterranean.
(DOCX)

S2 Table Biomarker scores of discrimination between the Mediterranean and habitual diet in the MedLey trial. Abbreviations: mol%, molar percent; RCT, randomised controlled trial; SD, standard deviation; wt%, weight percent; β-crypt., β-cryptoxanthin.
(DOCX)

S3 Table Percentages (numbers) of users of medications in the MedLey trial by randomised assignment at baseline (*n* = 152) and in the biomarker score derivation sample (*n* = 128). (DOCX)

S4 Table Medians (interquartile ranges) of the nutritional biomarker score of the Mediterranean diet in the MedLey trial post-intervention and in the EPIC-InterAct subcohort. Abbreviations: Hab, habitual; Med, Mediterranean; *n*, number of participants.
(DOCX)

S5 Table Nutritional biomarker score of the Mediterranean diet derived in the MedLey trial and incidence of type 2 diabetes in EPIC-InterAct: sensitivity analyses. Abbreviations: CI, confidence interval; EPIC, European Prospective Investigation into Cancer and Nutrition; HR, hazard ratio; MI, myocardial infarction; RCT, randomised controlled trial; SD, standard deviation.
(DOCX)

S6 Table Nutritional biomarker score of the Mediterranean diet derived in the MedLey trial and incidence of type 2 diabetes in EPIC-InterAct: associations per 1 standard deviation by categories of covariates. Abbreviations: CI, confidence interval; EPIC, European Prospective Investigation into Cancer and Nutrition; HR, hazard ratio; RCT, randomised controlled trial.
(DOCX)

S7 Table The score of self-reported Mediterranean diet. (DOCX)

S1 Fig Designs of the MedLey trial and the EPIC-InterAct case-cohort study and numbers of participants included in the analysis. (DOCX)

S2 Fig Differences in standardised means of nutritional biomarkers between the Mediterranean and habitual diet groups in the MedLey trial at 6 months. Abbreviations: AA, arachidonic acid; ALA, α-linolenic acid; DGLA, dihomo-γ-linolenic acid; DHA, docosahexaenoic acid; DPA, docosapentaenoic acid; EPA, eicosapentaenoic acid; FAs, fatty acids; LA, linoleic acid. Mixed linear models were used to estimate standardised differences after 6 months of partial-feeding intervention. Standardised values were calculated using baseline means and standard deviations of natural logarithm-transformed values of biomarkers. Horizontal error bars indicate 95% confidence intervals. Between 131–136 participants had non-missing biomarker data at 6 months.
(DOCX)

S3 Fig Calibration plots of the nutritional biomarker score for prediction of randomised assignment to 6 months of the Mediterranean diet (*n* = 67) versus continuation of habitual diet (*n* = 61) in the MedLey trial. Abbreviations: AUC, area under the curve; CITL, calibration-in-the-large; E:O, ratio of expected and observed outcomes.
(DOCX)

S1 Protocol Study protocol. (DOCX)

## Abbreviations

CI: confidence interval
EPIC: European Prospective Investigation into Cancer and Nutrition
HPLC: high-performance liquid chromatography
HR: hazard ratio
IQR: interquartile range
PAF: population attributable fraction
RCT: randomised controlled trial
SD: standard deviation
TC: total cholesterol
T2D: type 2 diabetes
