# Supplementary material for: A nutritional biomarker score of the Mediterranean diet and incident type 2 diabetes: Integrated analysis of data from the MedLey randomised controlled trial and the EPIC-InterAct case-cohort study
Source: PLoS Med. 2023 Apr 27;20(4):e1004221. doi: 10.1371/journal.pmed.1004221 (PMC10138823; doi:10.1371/journal.pmed.1004221)
Supplement: S1 Text — (DOCX) [file pmed.1004221.s003.docx]

**S1 Text.** Supplementary methods for “A nutritional biomarker score for adherence to the Mediterranean diet and incident type 2 diabetes: integrated analysis using data from the MedLey randomised controlled trial and the EPIC-InterAct case-cohort study”

*MedLey trial*

In the MedLey trial, individual nutritional biomarkers were pre-specified secondary outcomes. Derivation of the nutritional biomarker score was a post-hoc analysis which required additional methods beyond the original statistical analysis plan [1]. Mixed linear modelling with unstructured covariance under the intention-to-treat analysis was the pre-specified analytical approach for estimation of differences between the Mediterranean and habitual diet groups in continuous outcomes [1,2]. We used it to estimate the end-of-trial differences in the biomarker score, as well as individual biomarkers in order to facilitate the interpretation of the effects of intervention in the MedLey trial. The biomarker score and biomarkers were standardised (mean = 0, standard deviation = 1) using the baseline means and standard deviations. Use of medications was compared between the trial arms using the Chi-squared test. Multiplicative interactions between use of medications and the biomarker score were tested using logistic regression with randomised assignment as the outcome.

*Additional information on derivation of the biomarker score*

Ten-fold cross-validation was used in the elastic net regression models to select the λ penalties from a grid of 100 values per each α value [3]. The α values were tested in 0.1 increments between 0.5 and 0.9. Range favouring the lasso penalty was applied to enable selection of sufficiently parsimonious models for convergence of the post-selection unpenalised regressions given the sample size of the MedLey trial. Multivariable fractional polynomial logistic regression was used to evaluate the validity of fitting log-transformed linear biomarker terms under the log-linearity assumption in logistic regression [4].

Cross-validated C-statistic was used to evaluate the discriminatory performance between the two intervention arms in the MedLey trial. Cross-validation was implemented by randomly splitting the sample into 5 folds with sampling stratified by the randomised group assignment. Sequentially, 4 folds were used to re-estimate the coefficients for the biomarker score, followed by calculation of the C-statistic in the left-out fold. The cross-validated C-statistic was the average of the five C-statistic values calculated in this manner. Calibration was assessed by plotting a flexible curve of predicted versus observed probabilities of randomised assignment to the Mediterranean diet intervention based on cross-validated predictions from the biomarker score models [5].

We derived several secondary biomarker scores with the following alternative analytical decisions: changing the selection rate cut-off for inclusion of the predictors from 90% to 95% and 99% across repeated elastic net regression models, changing the unit of fatty acids from mol% to weight% and adjustment of biomarker score coefficients for use of medications.

*The score of self-reported Mediterranean diet*

The score of self-reported Mediterranean diet included assessment of the intake of nine components: (positively scored) vegetables, legumes, fruits and nuts, cereal, fish and seafood, olive oil, moderate alcohol use, and (negatively scored) meat and meat products, and dairy products [6]. The scoring cut-offs for food groups were based on tertiles of the EPIC-InterAct subcohort distributions of energy-standardised estimated intakes. Thus, each food group was assigned into a tertile category of 0, 1 or 2 for the adherence to each component and summed, producing a range between 0 and 18. The olive oil intake was scored as 0 in non-consumers, 1 for estimated energy-standardised intake below the median of subcohort consumers, and 2 for estimated intake at or above the median. For alcohol, the estimated intakes within sex-specific ranges of moderate consumption were scored as 2 points and the estimated intakes outside of these ranges were assigned 0 points (S7 Table). Calculation of the score was restricted to participants with estimated energy intakes of 800-4,000 kcal/day in men and 500-3,500 kcal/day in women.

*Discriminatory performance of the biomarker score between extremes of the score of self-reported Mediterranean diet*

We compared country-specific ≥90th percentile versus ≤10th percentile of the Mediterranean diet score estimated from self-report in the MedLey trial baseline sample (n = 42) and the EPIC-InterAct subcohort (n= 4,298). The discriminatory performance of the biomarker score for this comparison was evaluated using the C-statistic. In EPIC-InterAct, it was calculated based on a logistic regression model comprising of interactions between the biomarker score and dummy variables representing countries.

**References**

1. Davis CR, Bryan J, Hodgson JM, et al. A randomised controlled intervention trial evaluating the efficacy of an Australianised Mediterranean diet compared to the habitual Australian diet on cognitive function, psychological wellbeing and cardiovascular health in healthy older adults (MedLey study): Protocol paper. *BMC Nutr*. 2015;1(1):35. doi:10.1186/s40795-015-0033-7

2. Davis C, Hodgson J, Bryan J, et al. Older Australians Can Achieve High Adherence to the Mediterranean Diet during a 6 Month Randomised Intervention; Results from the Medley Study. *Nutrients*. 2017;9(6):534. doi:10.3390/nu9060534

3. Zou H, Hastie T. Regularization and variable selection via the elastic net. *J R Stat Soc Ser B*. 2005;67(2):301–20. doi:10.1111/j.1467-9868.2005.00503.x

4. Sauerbrei W, Meier-Hirmer C, Benner A, Royston P. Multivariable regression model building by using fractional polynomials: Description of SAS, STATA and R programs. *Comput Stat Data Anal.* 2006;50(12):3464–85. doi:10.1016/j.csda.2005.07.015

5. Nattino G, Lemeshow S, Phillips G, Finazzi S, Bertolini G. Assessing the Calibration of Dichotomous Outcome Models with the Calibration Belt. https://doi.org/101177/1536867X1801700414. 2018;17(4):1003–14. doi:10.1177/1536867X1801700414

6. Romaguera D, Guevara M, Norat T, et al. Mediterranean diet and type 2 diabetes risk in the European Prospective Investigation into Cancer and Nutrition (EPIC) study: the InterAct project. *Diabetes Care*. 2011;34(9):1913–8. doi:10.2337/dc11-0891 pmid:21788627
