# Supplementary material for: A nutritional biomarker score of the Mediterranean diet and incident type 2 diabetes: Integrated analysis of data from the MedLey randomised controlled trial and the EPIC-InterAct case-cohort study
Source: PLoS Med. 2023 Apr 27;20(4):e1004221. doi: 10.1371/journal.pmed.1004221 (PMC10138823; doi:10.1371/journal.pmed.1004221)
Supplement: S1 Table — Abbreviations: Hab, habitual; Med, Mediterranean. (DOCX) [file pmed.1004221.s004.docx]

**S1 Table.** Medians (25th, 75^th^ percentile) of circulating carotenoids and fatty acids: the MedLey trial post-intervention and the EPIC-InterAct subcohort at baseline

|  | MedLey trial | |  | EPIC-InterAct | | | | | | | |
| --- | --- | --- | --- | --- | --- | --- | --- | --- | --- | --- | --- |
|  | Hab-diet | Med-diet |  | France | Italy | Spain | UK | Netherlands | Germany | Sweden | Denmark |
|  | n=65 | n=68 |  | n=529 | n=1,910 | n=3,423 | n=1,230 | n=1,426 | n=1,890 | n=924 | n=1,981 |
| **Carotenoids, ng/mL*** |  |  |  |  |  |  |  |  |  |  |  |
| α-carotene | 20 | 35 |  | 98 | 42 | 26 | 57 | 33 | 47 | 50 | 41 |
|  | (9, 82) | (7, 187) |  | (65, 157) | (26, 74) | (16, 44) | (34, 92) | (19, 53) | (27, 85) | (28, 88) | (22, 77) |
| β-carotene | 726 | 998 |  | 419 | 251 | 144 | 246 | 197 | 248 | 227 | 163 |
|  | (440, 1029) | (637, 1588) |  | (278, 578) | (173, 365) | (94, 213) | (161, 350) | (126, 278) | (158, 395) | (150, 335) | (98, 266) |
| β-cryptoxanthin | 31 | 45 |  | 140 | 163 | 182 | 84 | 110 | 94 | 72 | 44 |
|  | (14, 80) | (14, 80) |  | (92, 225) | (90, 289) | (110, 302) | (48, 133) | (69, 182) | (57, 154) | (40, 124) | (24, 85) |
| Lycopene | 110 | 153 |  | 225 | 348 | 195 | 252 | 192 | 217 | 212 | 172 |
|  | (86, 150) | (102, 237) |  | (145, 314) | (261, 460) | (118, 285) | (157, 357) | (116, 303) | (143, 308) | (133, 301) | (104, 258) |
| Lutein & zeaxanthin | 513 | 426 |  | 234 | 273 | 194 | 143 | 151 | 162 | 146 | 123 |
|  | (370, 656) | (301, 664) |  | (175, 314) | (213, 353) | (148, 250) | (111, 192) | (111, 202) | (120, 212) | (113, 191) | (89, 168) |
|  |  |  |  |  |  |  |  |  |  |  |  |
| **Fatty acids, mol%**† |  |  |  |  |  |  |  |  |  |  |  |
| C14:0 | 0.46 | 0.39 |  | 0.41 | 0.36 | 0.28 | 0.38 | 0.40 | 0.39 | 0.40 | 0.39 |
|  | (0.39, 0.51) | (0.33, 0.45) |  | (0.34, 0.50) | (0.31, 0.42) | (0.23, 0.34) | (0.32, 0.45) | (0.33, 0.47) | (0.33, 0.47) | (0.34, 0.48) | (0.33, 0.46) |
| C16:0 | 28 | 27 |  | 30 | 30 | 29 | 30 | 30 | 30 | 31 | 31 |
|  | (27, 28) | (27, 28) |  | (29, 32) | (29, 31) | (28, 30) | (29, 31) | (29, 31) | (29, 31) | (30, 32) | (30, 32) |
| C18:0 | 12 | 12 |  | 14 | 14 | 15 | 14 | 14 | 14 | 14 | 14 |
|  | (11, 13) | (11, 12) |  | (14, 15) | (13, 15) | (14, 16) | (13, 15) | (13, 15) | (13, 15) | (13, 14) | (13, 14) |
| C15:0 | 0.29 | 0.28 |  | 0.27 | 0.21 | 0.17 | 0.23 | 0.24 | 0.22 | 0.22 | 0.20 |
|  | (0.25, 0.35) | (0.22, 0.32) |  | (0.23, 0.31) | (0.18, 0.25) | (0.14, 0.21) | (0.20, 0.27) | (0.21, 0.28) | (0.18, 0.27) | (0.18, 0.25) | (0.17, 0.24) |
| C17:0 | 0.41 | 0.41 |  | 0.49 | 0.42 | 0.44 | 0.42 | 0.40 | 0.39 | 0.39 | 0.39 |
|  | (0.38, 0.46) | (0.36, 0.44) |  | (0.44, 0.55) | (0.36, 0.46) | (0.38, 0.50) | (0.37, 0.47) | (0.36, 0.44) | (0.34, 0.43) | (0.36, 0.43) | (0.33, 0.43) |
| C20:0 | 0.15 | 0.15 |  | 0.13 | 0.13 | 0.12 | 0.15 | 0.14 | 0.12 | 0.14 | 0.12 |
|  | (0.13, 0.16) | (0.13, 0.16) |  | (0.12, 0.15) | (0.11, 0.14) | (0.11, 0.14) | (0.13, 0.18) | (0.12, 0.16) | (0.11, 0.14) | (0.12, 0.15) | (0.11, 0.14) |
| C22:0 | 0.39 | 0.36 |  | 0.24 | 0.21 | 0.22 | 0.26 | 0.28 | 0.20 | 0.23 | 0.22 |
|  | (0.33, 0.44) | (0.32, 0.41) |  | (0.21, 0.28) | (0.18, 0.24) | (0.19, 0.26) | (0.22, 0.30) | (0.24, 0.32) | (0.17, 0.23) | (0.21, 0.25) | (0.19, 0.25) |
| C24:0 | 1.07 | 1.02 |  | 0.25 | 0.22 | 0.22 | 0.23 | 0.26 | 0.20 | 0.21 | 0.21 |
|  | (0.94, 1.27) | (0.89, 1.15) |  | (0.22, 0.29) | (0.20, 0.25) | (0.19, 0.25) | (0.20, 0.26) | (0.23, 0.30) | (0.18, 0.23) | (0.19, 0.24) | (0.18, 0.23) |
| C18:3-n3 | 0.17 | 0.17 |  | 0.28 | 0.28 | 0.19 | 0.33 | 0.26 | 0.31 | 0.39 | 0.29 |
|  | (0.15, 0.19) | (0.15, 0.20) |  | (0.21, 0.39) | (0.20, 0.37) | (0.13, 0.28) | (0.23, 0.47) | (0.18, 0.38) | (0.24, 0.42) | (0.32, 0.51) | (0.23, 0.36) |
| C20:5-n3 | 1.46 | 1.79 |  | 1.07 | 0.71 | 0.86 | 1.10 | 0.85 | 0.98 | 1.30 | 1.59 |
|  | (0.96, 2.62) | (1.16, 2.29) |  | (0.79, 1.50) | (0.54, 0.90) | (0.60, 1.26) | (0.80, 1.56) | (0.62, 1.17) | (0.74, 1.32) | (0.99, 1.65) | (1.20, 2.26) |
| C22:5-n3 | 2.78 | 2.66 |  | 0.99 | 0.75 | 0.66 | 1.01 | 0.97 | 0.92 | 1.06 | 1.03 |
|  | (2.49, 3.10) | (2.37, 2.92) |  | (0.85, 1.14) | (0.66, 0.86) | (0.58, 0.75) | (0.87, 1.16) | (0.82, 1.09) | (0.80, 1.04) | (0.94, 1.20) | (0.91, 1.17) |
| C22:6-n3 | 5.4 | 5.8 |  | 4.8 | 3.5 | 4.6 | 4.1 | 3.4 | 3.6 | 4.1 | 4.7 |
|  | (4.4, 6.3) | (5.1, 6.3) |  | (4.0, 5.5) | (2.9, 4.1) | (3.9, 5.3) | (3.3, 5.0) | (2.8, 4.1) | (3.0, 4.3) | (3.4, 4.8) | (3.9, 5.5) |
| C18:2-n6c | 12 | 12 |  | 21 | 22 | 23 | 23 | 24 | 23 | 22 | 22 |
|  | (10, 12) | (11, 13) |  | (19, 23) | (20, 23) | (21, 25) | (21, 25) | (22, 26) | (21, 25) | (21, 24) | (20, 24) |
| C20:2 | 0.21 | 0.22 |  | 0.38 | 0.36 | 0.37 | 0.39 | 0.40 | 0.38 | 0.37 | 0.35 |
|  | (0.19, 0.24) | (0.21, 0.26) |  | (0.34, 0.42) | (0.33, 0.40) | (0.33, 0.41) | (0.35, 0.44) | (0.36, 0.45) | (0.34, 0.43) | (0.34, 0.42) | (0.32, 0.39) |
| C20:3-n6 | 1.4 | 1.5 |  | 3.1 | 3.7 | 3.0 | 3.2 | 3.3 | 3.1 | 3.1 | 2.8 |
|  | (1.3, 1.7) | (1.3, 1.7) |  | (2.5, 3.6) | (3.2, 4.2) | (2.5, 3.5) | (2.7, 3.7) | (2.8, 3.7) | (2.7, 3.6) | (2.7, 3.6) | (2.4, 3.2) |
| C20:4-n6 | 12.5 | 11.8 |  | 9.6 | 10.3 | 9.7 | 8.3 | 9.3 | 9.6 | 8.4 | 8.5 |
|  | (11.2, 13.4) | (11.3, 12.7) |  | (8.6, 10.9) | (9.1, 11.5) | (8.6, 11.0) | (7.3, 9.5) | (8.2, 10.4) | (8.5, 10.7) | (7.5, 9.4) | (7.5, 9.4) |
| C22:4 | 2.27 | 2.06 |  | 0.30 | 0.33 | 0.26 | 0.27 | 0.31 | 0.30 | 0.28 | 0.24 |
|  | (1.63, 2.88) | (1.69, 2.44) |  | (0.26, 0.35) | (0.29, 0.39) | (0.22, 0.31) | (0.23, 0.32) | (0.27, 0.36) | (0.26, 0.35) | (0.25, 0.31) | (0.21, 0.29) |
| C22:5-n6 | 0.28 | 0.25 |  | 0.22 | 0.28 | 0.18 | 0.16 | 0.21 | 0.21 | 0.16 | 0.13 |
|  | (0.21, 0.36) | (0.21, 0.28) |  | (0.18, 0.27) | (0.23, 0.33) | (0.15, 0.22) | (0.13, 0.20) | (0.16, 0.26) | (0.17, 0.25) | (0.13, 0.19) | (0.10, 0.16) |
| C16:1 | 0.41 | 0.39 |  | 0.44 | 0.46 | 0.32 | 0.49 | 0.48 | 0.53 | 0.51 | 0.58 |
|  | (0.34, 0.53) | (0.30, 0.50) |  | (0.35, 0.55) | (0.38, 0.60) | (0.26, 0.42) | (0.38, 0.63) | (0.38, 0.64) | (0.42, 0.70) | (0.42, 0.64) | (0.45, 0.76) |
| C17:1 | 0.11 | 0.09 |  | 0.03 | 0.07 | 0.05 | 0.04 | 0.04 | 0.07 | 0.06 | 0.07 |
|  | (0.08, 0.13) | (0.07, 0.11) |  | (0.02, 0.09) | (0.05, 0.10) | (0.04, 0.07) | (0.02, 0.08) | (0.02, 0.08) | (0.03, 0.11) | (0.04, 0.08) | (0.05, 0.10) |
| C18:1-n9c | 16.8 | 17.8 |  | 9.0 | 10.8 | 9.8 | 9.4 | 8.7 | 9.3 | 10.5 | 9.8 |
|  | (16.4, 17.4) | (16.8, 18.1) |  | (8.2, 10.0) | (9.7, 12.0) | (8.3, 11.2) | (8.4, 10.4) | (7.9, 9.7) | (8.6, 10.2) | (9.8, 11.3) | (9.0, 10.8) |
| C20:1 | 0.23 | 0.25 |  | 0.26 | 0.23 | 0.20 | 0.31 | 0.25 | 0.25 | 0.28 | 0.25 |
|  | (0.21, 0.25) | (0.23, 0.29) |  | (0.23, 0.29) | (0.20, 0.26) | (0.18, 0.23) | (0.27, 0.37) | (0.22, 0.29) | (0.21, 0.29) | (0.26, 0.32) | (0.20, 0.29) |
| C24:1 | 1.22 | 1.31 |  | 0.37 | 0.35 | 0.33 | 0.33 | 0.32 | 0.32 | 0.36 | 0.36 |
|  | (1.02, 1.35) | (1.18, 1.43) |  | (0.32, 0.42) | (0.30, 0.40) | (0.28, 0.38) | (0.28, 0.40) | (0.27, 0.38) | (0.28, 0.37) | (0.31, 0.40) | (0.31, 0.41) |
| C18:1-n9t | 0.10 | 0.10 |  | 0.17 | 0.13 | 0.13 | 0.40 | 0.37 | 0.18 | 0.35 | 0.20 |
|  | (0.08, 0.11) | (0.07, 0.11) |  | (0.12, 0.29) | (0.10, 0.18) | (0.10, 0.18) | (0.29, 0.56) | (0.26, 0.54) | (0.13, 0.24) | (0.25, 0.49) | (0.15, 0.27) |

Abbreviations: Hab – habitual; Med – Mediterranean

*Carotenoids were measured in serum in the MedLey trial and in plasma in EPIC-InterAct.

†Fatty acids were measured in erythrocytes in the MedLey trial and in plasma phospholipids in EPIC-InterAct. The denominator for the mol% unit was the sum of all fatty acids presented in this table.
