# Supplementary material for: A nutritional biomarker score of the Mediterranean diet and incident type 2 diabetes: Integrated analysis of data from the MedLey randomised controlled trial and the EPIC-InterAct case-cohort study
Source: PLoS Med. 2023 Apr 27;20(4):e1004221. doi: 10.1371/journal.pmed.1004221 (PMC10138823; doi:10.1371/journal.pmed.1004221)
Supplement: S2 Table — Abbreviations: mol%, molar percent; RCT, randomised controlled trial; SD, standard deviation; wt%, weight percent; β-crypt., β-cryptoxanthin. (DOCX) [file pmed.1004221.s005.docx]

**S2 Table.** Nutritional biomarker scores of discrimination between the Mediterranean and habitual diet in the MedLey trial*

| Biomarker† | Natural log mean (SD) | C-statistic after exclusion from the score with selection rate ≥ 90% and mol% fatty acids‡ | Interacting biomarker | Scoring coefficients by selection rate cut-off and the unit of fatty acids§ | | | |
| --- | --- | --- | --- | --- | --- | --- | --- |
|  |  |  |  | ≥ 90%, mol% | ≥ 95%, mol% | ≥ 99%, mol% | ≥ 90%, wt% |
| ß-carotene | 6.78 (0.81) | 0.86 | - |  |  |  |  |
|  |  |  | C18:1-n9c | 0.054 | 0.066 | 0.100 | 0.117 |
|  |  |  | C22:0 | -0.054 |  |  |  |
| β-crypt. | 3.55 (1.06) | 0.84 | C24:1 | 0.244 | 0.245 |  |  |
| Lycopene | 4.93 (0.69) | 0.86 | - |  |  |  |  |
|  |  |  | C22:6-n3 | 0.047 | 0.066 | 0.071 |  |
|  |  |  | C18:1-n9c | 0.045 |  |  |  |
| Lutein and zeaxanthin | 6.17 (0.50) | 0.82 | C20:1 | 0.166 | 0.174 | 0.276 | 0.250 |
| C15:0 | -1.28 (0.25) | 0.84 | ß-carotene | -0.043 | -0.048 | -0.112 |  |
| C17:0 | -0.91 (0.15) | 0.84 | ß-carotene | -0.089 | -0.104 |  |  |
| C18:0 | 2.47 (0.06) | 0.84 | C20:1 | 0.309 | 0.318 | 0.522 | 0.550 |
| C22:0 | -1.00 (0.19) | 0.84 | C24:1 | -1.423 | -1.478 | -2.855 | -3.762 |
| C24:0 | 0.04 (0.18) | 0.84 | - |  |  |  |  |
|  |  |  | C24:1 | -3.915 | -4.246 | -5.655 | 1.193 |
| C18:3-n3 | -1.76 (0.24) | 0.84 | C20:1 | -0.317 | -0.333 |  |  |
| C20:5-n3 | 0.49 (0.52) | 0.84 | C24:0 | -1.467 | -1.609 | -2.018 |  |
| C22:5-n3 | 1.00 (0.17) | 0.84 | C22:4-n6 | -0.495 | -0.497 |  |  |
| C22:6-n3 | 1.71 (0.19) | 0.85 | C17:1 | -0.128 | -0.122 |  |  |
| C18:2-n6 | 2.46 (0.14) | 0.84 | C20:1 | 0.292 | 0.297 |  |  |
| C20:4-n6 | 2.49 (0.13) | 0.84 | C20:1 | 0.228 | 0.219 | 0.445 | 0.395 |
| C22:5-n6 | -1.36 (0.28) | 0.85 | - |  |  |  |  |
|  |  |  | ß-carotene | -0.051 | 0.054 |  | -0.072 |
|  |  |  | Lycopene | -0.065 | -0.077 | -0.119 | -0.101 |
|  |  |  | C15:0 | 0.209 | 0.202 |  | 0.372 |
|  |  |  | C17:0 | 0.431 | 0.424 | 1.046 | 0.742 |
|  |  |  | C22:0 | 0.333 | 0.364 | 0.717 | 0.515 |
|  |  |  | C17:1 | 0.159 | 0.155 |  |  |
| C16:1 | -0.90 (0.36) | 0.84 | C24:0 | 0.813 | 0.918 |  |  |
| C17:1 | -2.33 (0.32) | 0.85 | - |  |  |  |  |
|  |  |  | ß-carotene | -0.045 | -0.050 | -0.082 | -0.085 |
| C18:1-n9c | 2.84 (0.06) | 0.86 | - | 3.247 | 3.589 | 4.218 | 4.052 |
| C20:1 | -1.41 (0.19) | 0.78 | - |  |  |  |  |
|  |  |  | C24:0 | 0.429 | 0.491 |  |  |
| C24:1 | 0.21 (0.19) | 0.84 | - |  |  |  |  |
|  |  |  | C22:5-n6 | -0.765 | -0.781 |  |  |
| C18:1-n9t | -2.40 (0.29) | 0.84 | C20:1 | -0.280 | -0.296 |  |  |
| Base odds |  |  |  | -10.463 | -10.933 | -14.276 | -13.893 |

Abbreviations: mol% - molar percent; RCT – randomised controlled trial; SD – standard deviation; wt% - weight percent; β-crypt. – β-cryptoxanthin

*n = 67 in the Mediterranean diet group and n = 61 in the continuation of habitual diet group

†Serum carotenoids were adjusted for total cholesterol using the residual method, re-scaled to the unadjusted mean, and expressed in ng/mL. Erythrocyte fatty acids were proportions of total fatty acids used in the analysis.

‡Values were estimated by calculating the score with omission of a given biomarker and its interaction terms and testing the discriminatory performance. The full score without omissions had a C-statistic of 0.91.

§Values are unstandardised coefficients of natural logarithm-transformed biomarkers from elastic net logistic regression models (on the log scale). Cross-validated logistic elastic net regression was repeated 1,000 times and predictors were included at pre-specified selection rate cut-offs. The primary pre-specified cut-off was ≥ 90%. The wt% fatty acids model was the same for the ≥ 90% and ≥ 95% cut-offs. C-statistic values ranged from 0.89-0.91, and 0.85-0.87 with 5-fold cross-validation.
