# Supplementary material for: A nutritional biomarker score of the Mediterranean diet and incident type 2 diabetes: Integrated analysis of data from the MedLey randomised controlled trial and the EPIC-InterAct case-cohort study
Source: PLoS Med. 2023 Apr 27;20(4):e1004221. doi: 10.1371/journal.pmed.1004221 (PMC10138823; doi:10.1371/journal.pmed.1004221)
Supplement: S3 Table — (DOCX) [file pmed.1004221.s006.docx]

**Table S3** Percentages (numbers) of users of medications in the MedLey trial by randomised assignment at baseline (n = 152) and in the biomarker score derivation sample (n = 128)*

| Medications | Habitual diet | Mediterranean diet | P-value† |
| --- | --- | --- | --- |
| Lipid-lowering |  |  |  |
| *Baseline sample* | 26.4 (19) | 32.5 (26) | 0.41 |
| *Biomarker score derivation sample‡* | 26.2 (16) | 37.3 (25) | 0.18 |
| *Interaction with biomarker score* |  |  | 0.46 |
| Anti-hypertensive |  |  |  |
| *Baseline sample* | 29.2 (21) | 40.0 (32) | 0.16 |
| *Biomarker score derivation sample* | 26.2 (16) | 43.3 (29) | 0.044 |
| *Interaction with biomarker score* |  |  | 0.93 |
| Anti-coagulants |  |  |  |
| *Baseline sample* | 12.5 (9) | 13.8 (11) | 0.82 |
| *Biomarker score derivation sample* | 11.5 (7) | 14.9 (10) | 0.57 |
| *Interaction with biomarker score* |  |  | 0.99 |
| Anti-reflux |  |  |  |
| *Baseline sample* | 11.1 (8) | 13.8 (11) | 0.62 |
| *Biomarker score derivation sample* | 9.8 (6) | 14.9 (10) | 0.39 |
| *Interaction with biomarker score* |  |  | 0.41 |
| Anti-osteoporotic |  |  |  |
| *Baseline sample* | 4.2 (3) | 6.3 (5) | 0.57 |
| *Biomarker score derivation sample* | 4.9 (3) | 7.5 (5) | 0.47 |
| *Interaction with biomarker score* |  |  | 0.99 |
| Other |  |  |  |
| *Baseline sample* | 19.4 (14) | 20.0 (16) | 0.93 |
| *Biomarker score derivation sample* | 21.3 (13) | 19.4 (13) | 0.79 |
| *Interaction with biomarker score* |  |  | 0.68 |

*Numbers of participants were 80 and 67 in the Mediterranean diet arm at baseline and in the biomarker score derivation sample at 6 months, respectively. The corresponding numbers were 72 and 61 in the habitual diet arm.

†P-values for between group differences in the proportions of users of medications were calculated using the chi-squared test. P-values for interaction refer to p-values for the coefficient of the interaction term between medication use and the biomarker score in logistic regression models predicting randomised assignment to diet groups in the biomarker score derivation sample at 6 months based on values of the biomarker score and the status of use of medications.

‡The biomarker score was derived after 6 months of the intervention with exclusion of participants who withdrew, had missing data or extreme values in nutritional biomarkers. All participants retained their baseline status of users or non-users of groupings of medications presented in the current table. Three participants per arm made changes to dosages of their baseline medication regimens or acutely introduced short-term medications (e.g., antibiotics).
