# Supplementary material for: A nutritional biomarker score of the Mediterranean diet and incident type 2 diabetes: Integrated analysis of data from the MedLey randomised controlled trial and the EPIC-InterAct case-cohort study
Source: PLoS Med. 2023 Apr 27;20(4):e1004221. doi: 10.1371/journal.pmed.1004221 (PMC10138823; doi:10.1371/journal.pmed.1004221)
Supplement: S4 Table — Abbreviations: Hab, habitual; Med, Mediterranean; n, number of participants. (DOCX) [file pmed.1004221.s007.docx]

**S4 Table.** Medians (25th, 75^th^ percentile) of the nutritional biomarker score of the Mediterranean diet in the MedLey trial post-intervention and in the EPIC-InterAct subcohort

| Biomarker score*** | MedLey trial | |  | EPIC-InterAct | | | | | | | |
| --- | --- | --- | --- | --- | --- | --- | --- | --- | --- | --- | --- |
|  | Hab-diet | Med-diet |  | France | Italy | Spain | UK | Netherlands | Germany | Sweden | Denmark |
|  | n=65 | n=68 |  | n=529 | n=1,910 | n=3,423 | n=1,230 | n=1,426 | n=1,890 | n=924 | n=1,981 |
|  |  |  |  |  |  |  |  |  |  |  |  |
| Standardised values | -0.25 | 1.53 |  | 0.40 | -0.30 | -0.35 | 0.54 | -0.02 | -0.30 | 0.71 | 0.57 |
|  | (-0.89, 0.35) | (0.89, 2.16) |  | (-0.22, 0.96) | (-0.83, 0.24) | (-1.02, 0.29) | (-0.15, 1.18) | (-0.60, 0.53) | (-0.89, 0.31) | (0.32, 1.15) | (-0.00, 1.11) |
| Linear predictions | -1.1 | 1.2 |  | -9.0 | -11.9 | -12.1 | -8.4 | -10.7 | -11.9 | -7.7 | -8.3 |
|  | (-2.0, -0.4) | (0.3, 2.0) |  | (-11.5, -6.7) | (-14.0, -9.7) | (-14.8, -9.4) | (-11.3, -5.8) | (-13.1, -8.5) | (-14.3, -9.4) | (-9.3, -5.9) | (-10.6, -6.1) |
| Med-/hab-diet probability | 0.24 | 0.76 |  | 0.00 | 0.00 | 0.00 | 0.00 | 0.00 | 0.00 | 0.00 | 0.00 |
|  | (0.12, 0.41) | (0.58, 0.88) |  | (0.00, 0.00) | (0.00, 0.00) | (0.00, 0.00) | (0.00, 0.00) | (0.00, 0.00) | (0.00, 0.00) | (0.00, 0.00) | (0.00, 0.00) |

Abbreviations: Hab – habitual; Med – Mediterranean; n – number of participants

*The biomarker score was derived as a discriminatory model between the Mediterranean and habitual diet in the MedLey randomised partial-feeding controlled trial using circulating carotenoids and fatty acids. The standardised values are study-specific. The linear predictions are the log odds and the Med-/Hab-diet probabilities are the predicted probabilities of the assignment to the Mediterranean or habitual diet arms of the MedLey trial.
