# Supplementary material for: A nutritional biomarker score of the Mediterranean diet and incident type 2 diabetes: Integrated analysis of data from the MedLey randomised controlled trial and the EPIC-InterAct case-cohort study
Source: PLoS Med. 2023 Apr 27;20(4):e1004221. doi: 10.1371/journal.pmed.1004221 (PMC10138823; doi:10.1371/journal.pmed.1004221)
Supplement: S5 Table — Abbreviations: CI, confidence interval; EPIC, European Prospective Investigation into Cancer and Nutrition; HR, hazard ratio; MI, myocardial infarction; RCT, randomised controlled trial; SD, standard deviation. (DOCX) [file pmed.1004221.s008.docx]

**S5 Table.** Nutritional biomarker score of the Mediterranean diet derived in the MedLey trial and incidence of type 2 diabetes in EPIC-InterAct*: sensitivity analyses

| Model | HR (95% CI) per 1 SD |
| --- | --- |
| Main result | 0.71 (0.65-0.77) |
| First 7 years of follow-up | 0.68 (0.60-0.77) |
| > 7 years of follow-up | 0.73 (0.65-0.81) |
| Excluding the first 2 years of follow-up | 0.71 (0.66-0.78) |
| Excluding participants with HbA1c > 48mmol/mol | 0.73 (0.68-0.80) |
| Excluding participants with prevalent cancer, MI or stroke | 0.71 (0.65-0.77) |
| Excluding outliers in nutritional biomarkers† | 0.69 (0.64-0.75) |
| Biomarker score from a single elastic net regression | 0.71 (0.66-0.77) |
| Biomarker score calculated using unpenalised coefficients | 0.77 (0.73-0.82) |
| Biomarker score derived with predictors’ selection rate cut-off 95% | 0.71 (0.65-0.77) |
| Biomarker score derived with predictors’ selection rate cut-off 99% | 0.74 (0.69-0.79) |
| Biomarker score derived with fatty acids as %weight instead of %mol | 0.73 (0.65-0.82) |
| Biomarker score derived with adjustment for use of medications |  |
| anti-hypertensive | 0.71 (0.65-0.77) |
| +lipid-lowering, anti-coagulants, anti-reflux, anti-osteoporotic, other | 0.70 (0.64-0.77) |
| Additional adjustment for use of medications in EPIC-InterAct subsets |  |
| Anti-hypertensive treatment (n = 5,870; 3,237 incident cases) |  |
| main multivariable model | 0.78 (0.72-0.85) |
| +anti-hypertensive treatment | 0.79 (0.73-0.85) |
| Lipid-lowering treatment (n = 2,656; 1,457 incident cases) |  |
| main multivariable model | 0.67 (0.60-0.75) |
| +lipid-lowering treatment | 0.66 (0.59-0.74) |
| Additional adjustment for components of the biomarker score:‡ |  |
| C18:1-n9c | 0.70 (0.65-0.76) |
| ß-carotene # C15:0 | 0.76 (0.71-0.81) |
| ß-carotene # C17:0 | 0.77 (0.72-0.83) |
| ß-carotene # C22:0 | 0.77 (0.72-0.83) |
| ß-carotene # C22:5-n6 | 0.72 (0.66-0.77) |
| ß-carotene # C17:1 | 0.71 (0.65-0.77) |
| ß-carotene # C18:1-n9c | 0.76 (0.70-0.81) |
| ß-cryptoxanthin # C24:1 | 0.75 (0.70-0.81) |
| Lycopene # C22:6-n3 | 0.72 (0.67-0.78) |
| Lycopene # C22:5-n6 | 0.70 (0.65-0.76) |
| Lycopene # C18:1-n9c | 0.71 (0.66-0.77) |
| Lutein & zeaxanthin # C20:1 | 0.73 (0.67-0.78) |
| C18:0 # C20:1 | 0.68 (0.62-0.74) |
| C15:0 # C22:5-n6 | 0.70 (0.64-0.75) |
| C17:0 # C22:5-n6 | 0.70 (0.65-0.75) |
| C22:0 # C22:5-n6 | 0.70 (0.65-0.75) |
| C22:0 # C24:1 | 0.78 (0.65-0.92) |
| C24:0 # C20:5-n3 | 0.59 (0.51-0.68) |
| C24:0 # C16:1 | 0.69 (0.63-0.75) |
| C24:0 # C20:1 | 0.72 (0.66-0.79) |
| C24:0 # C24:1 | 0.80 (0.67-0.97) |
| C18:3-n3 # C20:1 | 0.70 (0.65-0.76) |
| C22:5-n3 # C22:4 | 0.70 (0.65-0.76) |
| C22:6-n3 # C17:1 | 0.70 (0.64-0.76) |
| C18:2-n6c # C20:1 | 0.71 (0.65-0.77) |
| C20:4-n6 # C20:1 | 0.70 (0.64-0.76) |
| C22:5-n6 # C17:1 | 0.69 (0.64-0.75) |
| C22:5-n6 # C24:1 | 0.71 (0.65-0.77) |
| C18:1-n9t # C20:1 | 0.71 (0.66-0.77) |
| Simultaneous adjustment for all components of the biomarker score | 0.46 (0.30-0.72) |

Abbreviations: CI – confidence interval; EPIC – European Prospective Investigation into Cancer and Nutrition; HR – hazard ratio; MI – myocardial infarction; RCT – randomised controlled trial; SD – standard deviation

*The biomarker score was derived as a discriminatory model between the Mediterranean and habitual diet in the MedLey randomised partial-feeding controlled trial. Repeated elastic net regression models were used for variable selection, and predictors with selection rate ≥ 90% were included in the biomarker score. Circulating carotenoids and fatty acids were used to calculate the score as linear predictions from the discriminatory model. The multivariable adjusted model included the following covariates: age (as timescale), sex, recruitment centre, prevalent cancer, cardiovascular disease, hypertension and hyperlipidaemia; familial history of type 2 diabetes, smoking status (never, former, current smoker), physical activity index (inactive, moderately inactive, moderately active, active), seasonality (sine and cosine function of the day of the year), fasting status (<3, 3-6, >6 hours), current use of vitamin or mineral supplements, marital status (single, married or cohabiting, divorced or separated, widowed), educational attainment (none, primary school, technical or professional school, secondary school, post-secondary school education), current employment, body mass index and waist circumference, and in women, menopausal status (pre-, peri-, postmenopausal, bilateral oophorectomy), current hormone replacement therapy use. Hazard ratios were pooled from country-specific estimates. 22,202 participants with non-missing biomarker score data, including 9,453 incident cases of type 2 diabetes, were included in the analysis.

†Participants with nutritional biomarker values 4SDs above or below subcohort means in any of the component biomarkers of the biomarker score.

‡A hash mark denotes interaction. Biomarkers or biomarker-biomarker interactions were entered into the models as linear and squared terms. Only squared terms which were statistically significant when adjusting for individual components of the biomarker score were used in the analysis simultaneously adjusting for all components.
