# Supplementary material for: A nutritional biomarker score of the Mediterranean diet and incident type 2 diabetes: Integrated analysis of data from the MedLey randomised controlled trial and the EPIC-InterAct case-cohort study
Source: PLoS Med. 2023 Apr 27;20(4):e1004221. doi: 10.1371/journal.pmed.1004221 (PMC10138823; doi:10.1371/journal.pmed.1004221)
Supplement: S6 Table — Abbreviations: CI, confidence interval; EPIC, European Prospective Investigation into Cancer and Nutrition; HR, hazard ratio; RCT, randomised controlled trial. (DOCX) [file pmed.1004221.s009.docx]

**S6 Table.** Nutritional biomarker score of the Mediterranean diet derived in the MedLey trial and incidence of type 2 diabetes in EPIC-InterAct: associations per 1 standard deviation by categories of covariates*

| Covariate category | Multiply imputed analysis | | |  | Complete-case analysis | | |
| --- | --- | --- | --- | --- | --- | --- | --- |
|  | n† | HR (95% CI) | p_interaction_‡ |  | n | HR (95% CI) | p_interaction_‡ |
| Main result | 22,202 | 0.71 (0.65-0.77) |  |  | 15,430 | 0.70 (0.64-0.76) | - |
| Dietary supplements |  |  |  |  |  |  |  |
| Non-users | 13,819 | 0.69 (0.62-0.75) |  |  | 9,816 | 0.67 (0.61-0.74) |  |
| Users | 8,383 | 0.76 (0.69-0.84) | 0.21 |  | 5,614 | 0.75 (0.66-0.85) | 0.03 |
| Age at baseline, years |  |  |  |  |  |  |  |
| <45 | 4,234 | 0.54 (0.42-0.69) |  |  | 3,174 | 0.52 (0.38-0.70) |  |
| 45-60 | 12,892 | 0.74 (0.69-0.80) |  |  | 8,884 | 0.72 (0.65-0.80) |  |
| >60 | 5,076 | 0.74 (0.67-0.82) | 0.02 |  | 3,372 | 0.74 (0.65-0.84) | 0.01 |

Abbreviations: CI – confidence interval; EPIC – European Prospective Investigation into Cancer and Nutrition; HR – hazard ratio; RCT – randomised controlled trial

*The biomarker score was derived as a discriminatory model between the Mediterranean and habitual diet in the MedLey randomised partial-feeding controlled trial. Circulating carotenoids and fatty acids were used to calculate the score as linear predictions from the discriminatory model. Hazard ratios were pooled from country-specific estimates. Models were adjusted for: age (as timescale for effect modification by supplement use), sex, recruitment centre, prevalent cancer, cardiovascular disease, hypertension and hyperlipidaemia; familial history of type 2 diabetes, smoking status (never, former, current smoker), physical activity index (inactive, moderately inactive, moderately active, active), seasonality (sine and cosine function of the day of the year), fasting status (<3, 3-6, >6 hours), current use of vitamin or mineral supplements, marital status (single, married or cohabiting, divorced or separated, widowed), educational attainment (none, primary school, technical or professional school, secondary school, post-secondary school education), current employment, body mass index (BMI) and waist circumference, and in women, menopausal status (pre-, peri-, postmenopausal, bilateral oophorectomy), current hormone replacement therapy use. Presence of interaction was also evaluated for sex, BMI, seasonality, fasting status, physical activity and smoking status (p_interaction_ values > 0.05).

†Numbers of participants by use of dietary supplements in multiply imputed analysis are mid-point values between the smallest and the largest values in the imputation datasets.

‡Interaction p values for age are based on continuous-by-continuous interaction terms between age and biomarker score.
