# Supplementary material for: A nutritional biomarker score of the Mediterranean diet and incident type 2 diabetes: Integrated analysis of data from the MedLey randomised controlled trial and the EPIC-InterAct case-cohort study
Source: PLoS Med. 2023 Apr 27;20(4):e1004221. doi: 10.1371/journal.pmed.1004221 (PMC10138823; doi:10.1371/journal.pmed.1004221)
Supplement: S7 Table — (DOCX) [file pmed.1004221.s010.docx]

**S7 Table.** The score of self-reported Mediterranean diet

| Component, g/1,000 kcal | Range of points† | Minimum points | Maximum points |
| --- | --- | --- | --- |
|  |  |  |  |
| Vegetable | 0-2 | <57.6 | >100.3 |
| Legumes | 0-2 | <0.49 | >6.37 |
| Fruits and nuts | 0-2 | <66.0 | >133.8 |
| Cereals | 0-2 | <81.3 | >113.5 |
| Fish and seafood | 0-2 | <9.48 | >20.45 |
| Meat and meat products | 0-2 | >59.8 | <40.8 |
| Dairy | 0-2 | >194 | <102 |
| Olive oil | 0-2 | Non-consumers | >6.85 |
| Ethanol (g/day) | 0 or 2 | Intake outside of ranges for maximum points | Men: 10-50  Women: 5-25 |

*Cut-offs for minimum and maximum points for estimated intakes of foods were established in the EPIC-InterAct subcohort based on distributions of estimated intakes in this study. The same cut-offs were applied when calculating the score in the MedLey trial for assessment of self-reported adherence to the intervention.

†Integer points were used. One point was assigned for estimated intakes between cut-offs for minimum and maximum points, except for ethanol for which either 0 or 2 points were assigned.
