# Supplementary material for: A nutritional biomarker score of the Mediterranean diet and incident type 2 diabetes: Integrated analysis of data from the MedLey randomised controlled trial and the EPIC-InterAct case-cohort study
Source: PLoS Med. 2023 Apr 27;20(4):e1004221. doi: 10.1371/journal.pmed.1004221 (PMC10138823; doi:10.1371/journal.pmed.1004221)
Supplement: S1 Fig — (DOCX) [file pmed.1004221.s011.docx]

**EPIC-InterAct**

**MedLey trial**

**EPIC cohort participants eligible for inclusion (n=340,234)**

**Assessed for eligibility (n=210)**

**Excluded (n=44)**

- inclusion criteria not met (n=31)
- refusal to participate (n=6)
- unavailable for study visits (n=7)

**Baseline**

**Case-cohort assembly**

**Analysis**

**Follow-up**

**Analysis**

**Exclusions (n=681)**

- prevalent diabetes (n=548)
- uncertain diabetes status (n=133)

Overlapping T2D cases (n=564)

Overlapping T2D cases (n=778)

**Verified cases (n=9,453)**

**Biomarker score (n=128)**

**Verified cases (n=12,403)**

**Exclusions (n=5,525)**

- Denmark, due to logistical reasons (n=2,577)
- non-diabetic (n=838)
- prevalent diabetes (n=421)
- uncertain diabetes status (n=1,689)

**Ascertained T2D cases (n=17,928)**

**Random subcohort**

**(n=16,835)**

**Completed intervention (n=67)**

- biomarkers unavailable (n=2)
- excluded due to extreme biomarker concentrations (n=4)
- included (n = 61)

**Completed intervention (n=70)**

- biomarkers unavailable (n=2)
- excluded due to extreme biomarker concentrations (n=1)
- included (n = 67)

**Drop-outs (n=7)**

- health reasons not related to trial participation (n=1)
- family/personal issues (n=2)
- other commitments (n=1)

**Drop-outs (n=12)**

- non-compliance (n=2)
- health reasons not related to trial participation (n=3)
- family/personal issues (n=4)
- other commitments (n=1)

**Habitual diet (n=81)**

- withdrew (n=9)
- commenced (n=72)

**Mediterranean diet (n=85)**

- withdrew (n=5)
- commenced (n=80)

**Randomised (n=166)**

**Subcohort (n=16,154)**

**Exclusions (n=5,577)**

- Malmö, Sweden, carotenoids not assayed (n=3,556)
- missing biomarkers in participants from other centres (n=2,021)

**Subcohort (n=13,313)**

**S1 Fig.** Designs of the MedLey trial and the EPIC-InterAct case-cohort study and numbers of participants included in the analysis
