# Supplementary material for: A nutritional biomarker score of the Mediterranean diet and incident type 2 diabetes: Integrated analysis of data from the MedLey randomised controlled trial and the EPIC-InterAct case-cohort study
Source: PLoS Med. 2023 Apr 27;20(4):e1004221. doi: 10.1371/journal.pmed.1004221 (PMC10138823; doi:10.1371/journal.pmed.1004221)
Supplement: S2 Fig — Abbreviations: AA, arachidonic acid; ALA, α-linolenic acid; DGLA, dihomo-γ-linolenic acid; DHA, docosahexaenoic acid; DPA, docosapentaenoic acid; EPA, eicosapentaenoic acid; FAs, fatty acids; LA, linoleic acid. Mixed linear models were used to estimate standardised differences after 6 months of partial-feeding intervention. Standardised values were calculated using baseline means and standard deviations of natural logarithm-transformed values of biomarkers. Horizontal error bars indicate 95% confidence intervals. Between 131–136 participants had non-missing biomarker data at 6 months. (DOCX) [file pmed.1004221.s012.docx]

**
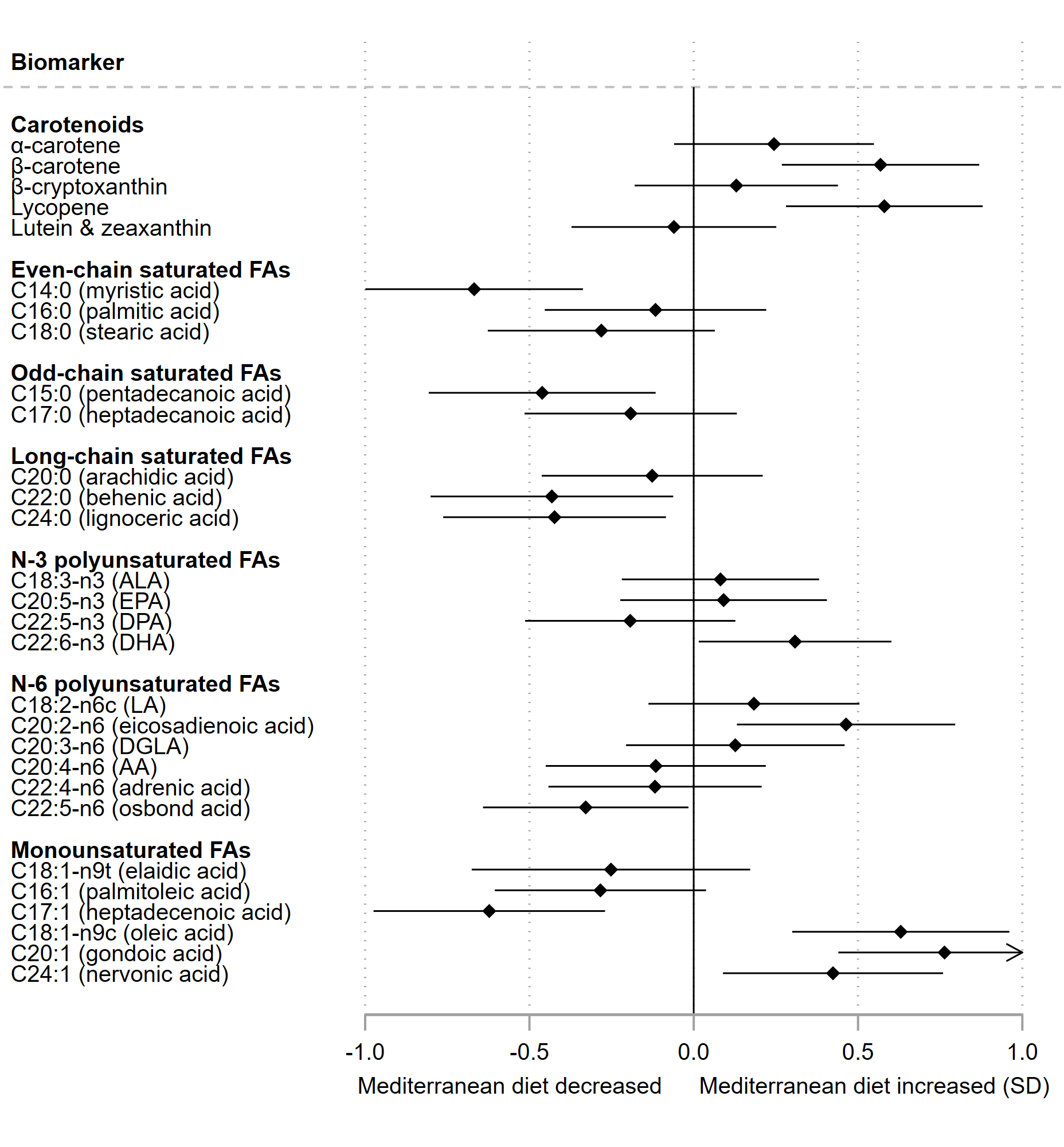
**

**S2** **Fig.** Differences in standardised means of nutritional biomarkers between the Mediterranean and habitual diet groups in the MedLey trial at 6 months

Abbreviations: AA – arachidonic acid; ALA – α-linolenic acid; DGLA – dihomo-γ-linolenic acid; DHA – docosahexaenoic acid; DPA – docosapentaenoic acid; EPA – eicosapentaenoic acid; FAs – fatty acids; LA – linoleic acid

Mixed linear models were used to estimate standardised differences after 6 months of partial-feeding intervention. Standardised values were calculated using baseline means and standard deviations of natural logarithm-transformed values of biomarkers. Horizontal error bars indicate 95% confidence intervals. Between 131-136 participants had non-missing biomarker data at 6 months.
