# Supplementary material for: A nutritional biomarker score of the Mediterranean diet and incident type 2 diabetes: Integrated analysis of data from the MedLey randomised controlled trial and the EPIC-InterAct case-cohort study
Source: PLoS Med. 2023 Apr 27;20(4):e1004221. doi: 10.1371/journal.pmed.1004221 (PMC10138823; doi:10.1371/journal.pmed.1004221)
Supplement: S3 Fig — Abbreviations: AUC, area under the curve; CITL, calibration-in-the-large; E:O, ratio of expected and observed outcomes. (DOCX) [file pmed.1004221.s013.docx]

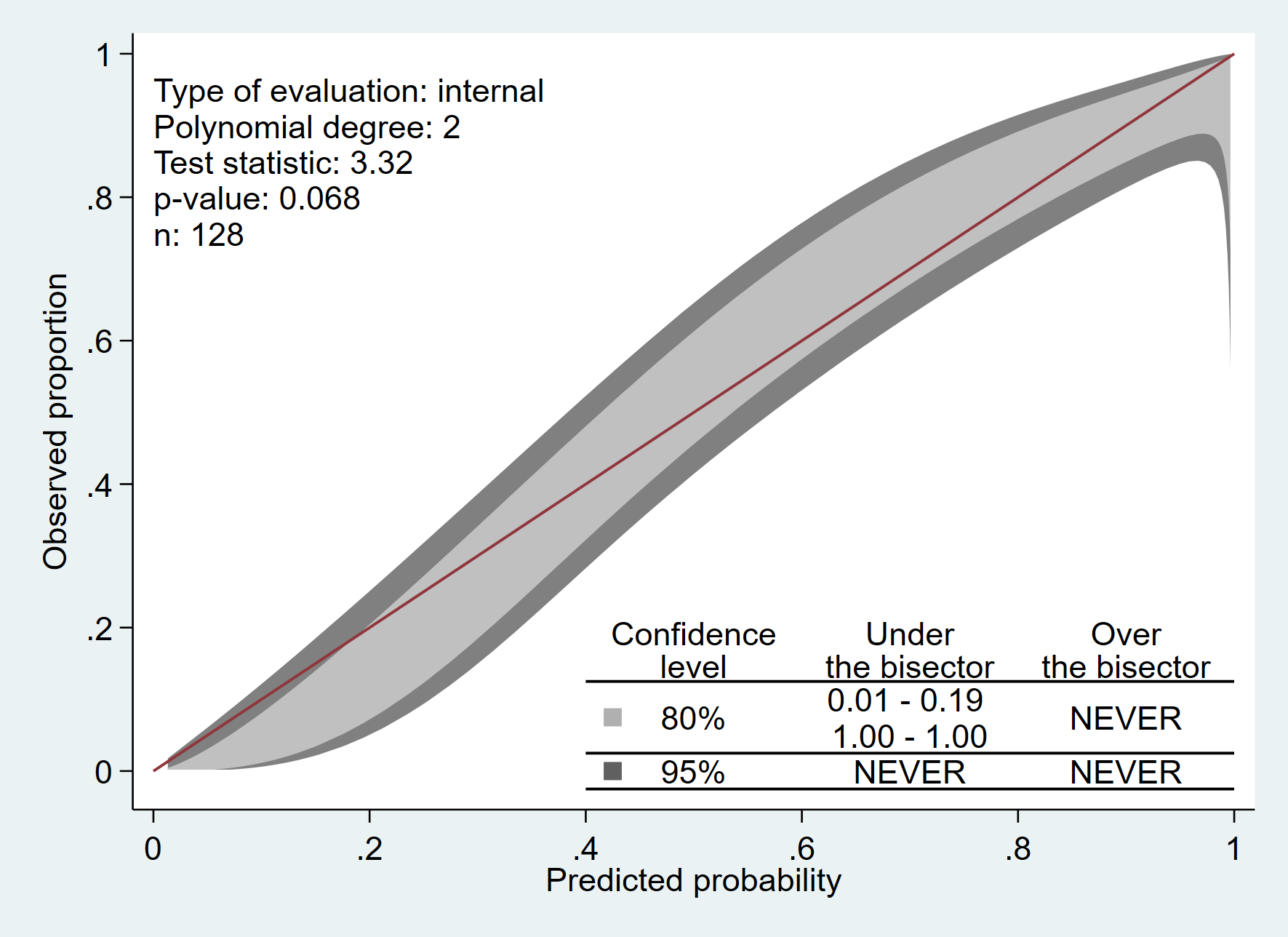


A. Calibration belt plot


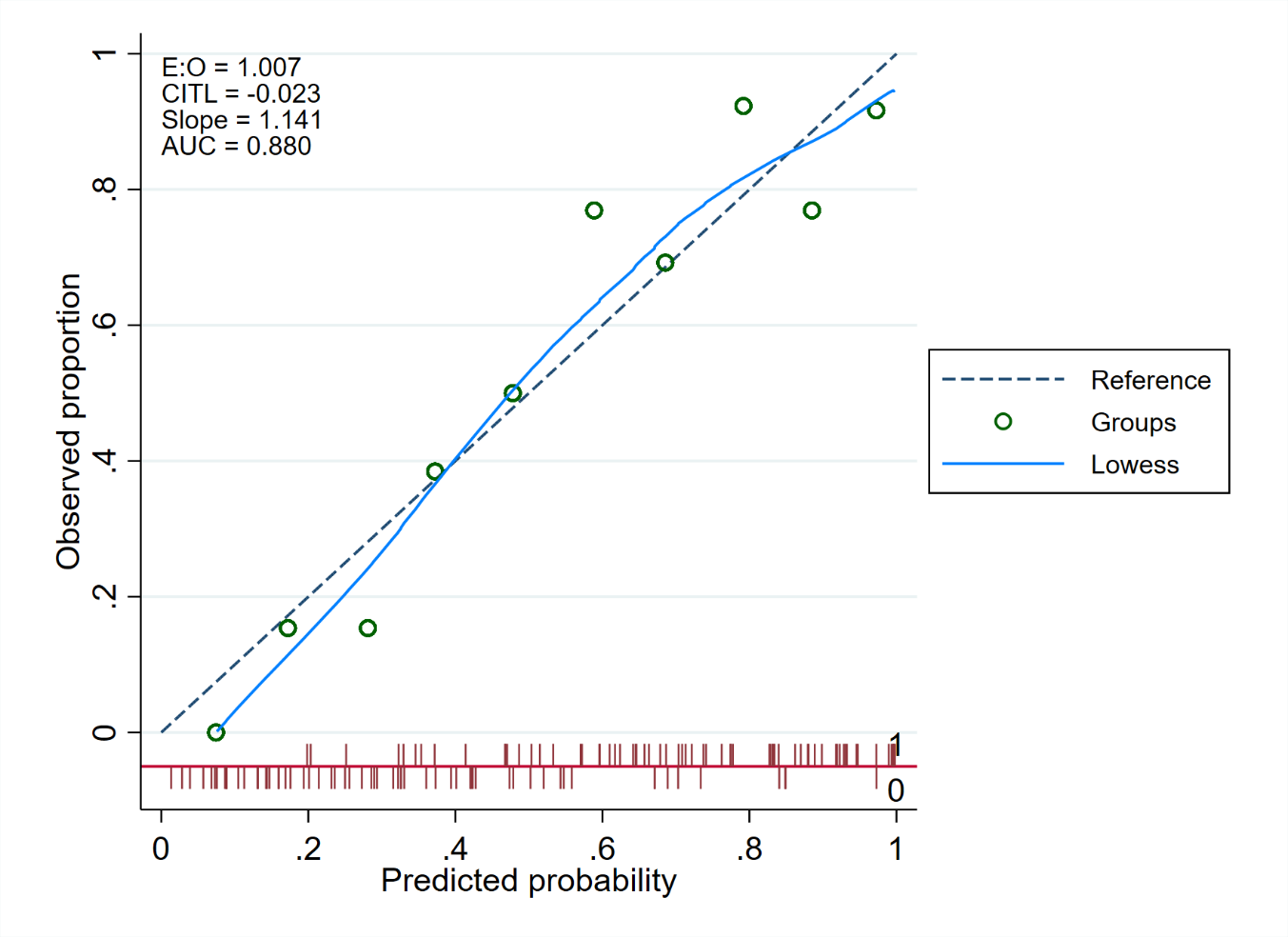
B. Calibration plot with a lowess smoother and 10-fold grouping of study participants.

**S3** **Fig.** Calibration plots of the nutritional biomarker score for prediction of randomised assignment to 6 months of the Mediterranean diet (n = 67) versus continuation of habitual diet (n = 61) in the MedLey trial

Abbreviations: AUC – area under the curve; CITL – calibration-in-the-large; E:O – ratio of expected and observed outcomes
