# Supplementary material for: A nutritional biomarker score of the Mediterranean diet and incident type 2 diabetes: Integrated analysis of data from the MedLey randomised controlled trial and the EPIC-InterAct case-cohort study
Source: PLoS Med. 2023 Apr 27;20(4):e1004221. doi: 10.1371/journal.pmed.1004221 (PMC10138823; doi:10.1371/journal.pmed.1004221)
Supplement: S1 Protocol — (DOCX) [file pmed.1004221.s014.docx]

**Title:** Protocols for the manuscript “A nutritional biomarker score of the Mediterranean diet and incident type 2 diabetes: integrated analysis of data from the MedLey randomised controlled trial and the EPIC-InterAct case-cohort study”

**Background:** The statistical analysis plan for this study was covered by two protocol documents written prior to establishing a collaboration between the EPIC-InterAct and MedLey studies. These documents are broader in their scope than the current analysis alone, and not all described analyses or named studies are included in the current manuscript.

**Protocol 1** (Pages 2-5 of this document) describes the joint analysis of the MedLey trial and EPIC-InterAct, for which we planned to use a publicly deposited dataset of the MedLey trial (<https://doi.org/10.3390/nu9060534>). The specific dataset did not include most individual fatty acids, but rather groupings of major fatty acids (e.g., sum of saturated fatty acids).

**Protocol 2** (Pages 6-9) describes an initial idea of the EPIC-InterAct investigators to derive nutritional biomarker scores of the Mediterranean diet and other dietary patterns internally based on prediction of self-reported dietary intakes from concentrations of all nutritional biomarkers measured in the cohort. The description of prospective analyses of associations between the biomarker scores and risk of type 2 diabetes was applied to the current analysis.

**Amendments** (Pages 10-11) describe further analytical and data presentation decisions for the current manuscript undertaken upon establishing a collaboration of EPIC-InterAct with the MedLey trial with full biomarker data sharing.

**References** for all documents are available on Page 12.

**Protocol 1 (20/07/2020):**

**Proposal of analysis: investigation of utility of nutritional biomarkers to assess adherence to Mediterranean diet. Longitudinal analysis of MedLey randomised controlled trial and longitudinal and cross-sectional analyses in European Prospective Investigation into Cancer and Nutrition (EPIC-Norfolk and EPIC-InterAct studies)**

**Brief description of studies:**

MedLey study is a two-arm parallel randomised controlled trial (RCT) of 137/166 (completed/enrolled) Australians aged ≥ 65 years [1]. Conducting a 6-month intervention, this RCT compared the effect of Mediterranean diet advice and provision of its key foods with the effect of a habitual diet on serum carotenoids, erythrocyte fatty acids and urinary electrolytes. As a measure of compliance to the intervention, self-reported adherence to Mediterranean diet was assessed using a 3-day weighed food diary at months 0, 2 and 4. Biomarkers were measured at months 0, 3 and 6. Raw data were made publicly available at: <https://doi.org/10.3390/nu9060534>.

EPIC-InterAct is a multi-country case-cohort study of type 2 diabetes nested within eight European countries of the European Prospective Investigation into Cancer Study (EPIC) [2]. EPIC-InterAct allows a large-scale epidemiological analysis of approximately twelve thousand incident cases of type 2 diabetes and sixteen thousand participants in the subcohort. Most participants had baseline measurements of plasma carotenoids and phospholipid fatty acids. Habitual diet was assessed using food frequency questionnaires (FFQ) or diet histories.

EPIC-Norfolk, one of the cohorts of EPIC, is a population-based cohort study in East of England [3]. Subsets of participants had baseline and follow-up measurements of plasma carotenoids, plasma phospholipid, and erythrocyte fatty acids, and 24-h and spot urinary sodium and potassium. Habitual diet was assessed at baseline and follow-up using FFQ and 7-day food diary.

**Background:**

Ongoing work in EPIC-InterAct and EPIC-Norfolk aims to derive biomarker scores of habitual adherence to Mediterranean diet using combinations of circulating carotenoids, fatty acids and other nutritional biomarkers. Briefly, cross-validated penalised regression has been modelled to predict Mediterranean diet scores based on nutritional biomarkers. Mediterranean diet scores were calculated based on self-reported diet, using responses to country-specific instruments for dietary assessment. Linear combinations of all available biomarkers were considered, alongside models incorporating first order interactions between biomarkers. Both country-specific and subcohort-wide biomarker scores were developed.

Assessment of external validity of the biomarker scores in a trial setting is desirable. MedLey study dataset includes some of the biomarkers measured in EPIC-InterAct and EPIC-Norfolk which allows for such external validation. The table below shows the overlap between nutritional biomarkers measured in the three studies.

Development of a biomarker of intervention with Mediterranean diet is also of interest. Such a biomarker would have applications in assessment of adherence to the dietary intervention in a clinical setting as well as a non-clinical population-based setting. MedLey trial allows to assess whether or not a specific combination of nutritional biomarkers can differentiate between intervention with Mediterranean diet and habitual diet. The resultant classification model from MedLey trial can be examined for its external validity for a population-based setting by evaluating EPIC-InterAct and EPIC-Norfolk studies.

| **Table** Availability of nutritional biomarker data in MedLey trial and overlap with EPIC-Interact and EPIC-Norfolk studies | | |
| --- | --- | --- |
| MedLey | EPIC-InterAct | EPIC-Norfolk |
|  |  |  |
| **Serum carotenoids** | **Plasma carotenoids** | **Plasma carotenoids** |
| α-carotene, β-carotene, β-cryptoxanthin, lycopene, sum of lutein and zeaxanthin | all available | all available |
|  |  |  |
| **Erythrocyte fatty acids** | **Plasma phospholipid fatty acids** | **Plasma phospholipid and erythrocyte fatty acids** |
| total SFA | sum of individual SFAs | sum of individual SFAs |
| total TFA | sum of individual TFAs | sum of individual TFAs |
| MUFA-n9 | sum of individual MUFA-n9 | sum of individual MUFA-n9 |
| MUFA-n7 | C17:1 | sum of individual MUFA-n7 |
| MUFA-other | sum of remaining MUFAs | sum of remaining MUFAs |
| individual n3 PUFAs: C18:3n3, C20:5n3, C22:5n3, C22:6n3, C20:4n-6 | all available | all available |
| individual n6 PUFAs: C18:2n6, C20:4n6 | all available | all available |
| PUFA n6-other | sum of remaining n6 PUFAs | sum of remaining n6 PUFAs |
|  |  |  |
| **24-h urinary electrolytes** |  |  |
| sodium, potassium, calcium, magnesium | none available | sodium, potassium |

Abbreviations: MUFA – monounsaturated fatty acids; PUFA – polyunsaturated fatty acids; SFA – saturated fatty acids; TFA – trans-fatty acids

Taking advantage of the overlap in measured biomarkers between MedLey trial and EPIC-InterAct, we propose to (i) validate EPIC-InterAct biomarker scores in MedLey trial and (ii) develop a biomarker score of clinical intervention assignment in the trial and evaluate whether it can discriminate between high and low adherence to Mediterranean diet in the cross-sectional setting of EPIC-InterAct.

**Statistical analysis plan:**

**1. Validation of biomarker scores developed in EPIC-InterAct and EPIC-Norfolk studies.**

Derivation of biomarker scores in EPIC-InterAct subcohort and EPIC-Norfolk will be repeated using the reduced set of biomarkers available in MedLey trial. In EPIC-InterAct it will be undertaken in 7 country-specific datasets and one subcohort-wide dataset (pooled dataset of the 7 countries). In each of these 8 datasets, 4 scores will be developed based on combinations of (i) adjusted and unadjusted analysis and (ii) presence or absence of interactions. Overall, 32 biomarker scores will be available. Subcohort-wide biomarker scores will be considered primary outcomes.

Analogous analysis will be undertaken in EPIC-Norfolk, adding further 4 scores. Additionally, we will repeat the analysis in EPIC-Norfolk using the 7-day food diary to estimate the Mediterranean diet score.

Linear mixed effects models will be used to assess the effect of intervention on standardized differences in biomarker scores between the intervention and control group. To reduce the risk of false-positive inference, we will apply false-discovery rate (FDR) correction. We will assume high dependence of different biomarker scores developed within each country or within the whole subcohort. Between-country differences in biomarker scores will be assumed to be driven largely by different data-generating processes. The country of derivation will be considered as a considerably larger source of variation within the pool of all available biomarker scores than the level of adjustment or presence of interactions in the analysis. Thus, FDR correction will be applied separately within groups of biomarker scores defined by the level of adjustment or presence of interactions. Within each of these groups, 9 biomarker scores (7 country-specific and one subcohort wide from EPIC-InterAct, and one from EPIC-Norfolk) will be tested with FDR correction.

Additionally, we will quantify the effects of change in self-reported adherence to Mediterranean diet in MedLey trial (using InterAct scoring cut-offs) on change in biomarker scores. Also, correlations between self-reported adherence and biomarker scores will be calculated cross-sectionally.

**2. Biomarker score of clinical intervention assignment**

This analysis will aim to evaluate whether combinations of nutritional biomarkers can discriminate between the Mediterranean and habitual diet in MedLey trial at 6 months. We will adapt to a binary outcome the methods for evaluation of nutritional biomarkers established in the Women’s Health Initiative feeding trial [4].

Natural-log transformation will be applied to biomarkers to improve normality of distributions. Values outside of 25^th^ percentile minus 3 times the interquartile range (IQR) or 75^th^ percentile plus 3 times the IQR will be removed from the analysis. Logistic regression will be used to develop the biomarker score. Backward selection procedure based on Bayesian information criterion will be used to remove non-informative biomarkers. Multivariable fractional polynomial logistic regression will be used to evaluate the validity of fitting ln-transformed linear terms under log-linearity assumption. Any identified nonlinearities will be included in the logistic regression model. Assumption of no interactions between biomarkers will be evaluated using elastic net logistic regression with first order interactions between biomarkers. Bootstrap-enhanced stability selection will be used to identify consistently selected interactions [5]. Any interactions selected in ≥ 95% of bootstrap samples will be included in the logistic regression model.

Five-fold cross-validated C-statistic will be the primary measure of model performance. We will also calculate net reclassification index and sensitivity and specificity in a clinically relevant scenario.

The analyses will be undertaken using (i) full biomarker data, (ii) excluding urinary calcium and magnesium and (iii) excluding all urinary electrolytes. It will allow to assess the added value of 24-h urine collection to blood-based biomarkers and result in biomarker scores applicable to EPIC-Norfolk (ii) and EPIC-InterAct (iii).

We will test whether the biomarker scores derived in MedLey trial can be used in EPIC-InterAct and EPIC-Norfolk (i) as a measure of degree of adherence to Mediterranean diet and (ii) to discriminate between high and low adherence to Mediterranean diet. The former aim will be addressed by linear regression of the trial-derived biomarker score and Mediterranean diet score based on dietary self-report. High and low adherence ranges will be defined by using the statistics in MedLey trial. To define the high adherence, we will use the median of the Mediterranean diet score +/- 1 point in the intervention group of MedLey trial. To define the low adherence, we will use the corresponding median +/- 1 point in the habitual diet group. Logistic regression and c-statistic will be used to evaluate discrimination between the two groups in EPIC studies. Net reclassification improvement will be estimated by analysing a predictive capability of the biomarker score in comparison to an intercept-only model and a multivariable-adjusted model including variables readily available in a clinical setting (age, sex, recruitment sites, and smoking status).

We will consider analysing prospective associations between the biomarker scores and type 2 diabetes (EPIC-InterAct) and cardiovascular endpoints (EPIC-Norfolk).

**Protocol 2 (12/04/2019):**

**Protocol:** The development of nutritional biomarker scores as indicators of dietary quality assessed by predefined dietary patterns, and evaluation of their association with and predictive ability for type 2 diabetes incidence

**Proposer**: Jakub Sobiecki

**Affiliation**: MRC Epidemiology Unit

**Working group participants**: Nita Forouhi, Marcela Guevara, Fumiaki Imamura, Albert Koulman, Matthias Schulze, Stephen Sharp

**Research question:** Nutritional biomarkers have been used to measure nutrient intake or status primarily on single biomarker-single nutrient basis. The use of multiple circulating biomarkers to assess overall dietary quality is rarely evaluated. Thus, we hypothesise that concurrent use of a wide range of biomarkers could reflect levels a specific dietary pattern. The expected increase in precision of capturing dietary exposure would increase the statistical power to detect diet-disease associations and also enable the evaluation of possible effect-modification of the association by genetic variation in susceptibility to obesity, insulin resistance or T2D. The InterAct study has measured self-reported habitual diet, gene variants, and a variety of nutritional biomarkers including circulating vitamin C, carotenoids, 25(OH)D and phospholipid fatty acids, and nutrition-related biomarkers such as calcium, magnesium and iron status markers. Thus, it provides a unique opportunity to develop nutritional biomarker scores of specific dietary patterns. The association of these scores with incident type 2 diabetes (T2D) could then be evaluated as a means of assessing predictive criterion validity.

**Objectives**

**(1)** To evaluate whether a combination of nutritional biomarker variables could be used to develop biomarker scores predictive of dietary patterns.

**(2)** To evaluate the association of the biomarker scores with T2D incidence.

**(3)** To evaluate the predictive ability of the biomarker scores for T2D incidence.

**Main exposures:**

1. Nutritional biomarker score(s) developed by using circulating vitamin C, carotenoids (α-carotene, β-carotene, β-cryptoxanthin, lycopene, lutein, zeaxanthin), and phospholipid fatty acids (individual and groups of fatty acids to include marine n-3 fatty acids [EPA+DHA+DPA], ALA, LA (n-6 PUFA), odd-chain SFA, even-chain SFA, very-long-chain SFA, MUFA and trans fatty acids). We will also include nutrition-related biomarkers: circulating 25(OH)D, 25(OH)D_3_-epimer (D_2_ and D_3_-non-epimer will not be used due to high rates of missing data [6]), magnesium, calcium, and iron status markers. As specified in the analysis of individual fatty acids [7], fatty acids with concentrations < 0.05 mol% will not be used.

2. Self-reported dietary factors including major food groups and predefined dietary patterns [Mediterranean diet score (MDS), Dietary Approaches to Stop Hypertension (DASH), Alternative-Healthy Eating Index 2010 (aHEI)].

**Main potential confounding factors:**

**1. Association of biomarkers with dietary pattern scores:** Country, centre, age, sex, BMI, waist circumference, education, physical activity, smoking status, medication use, season of blood draw, total cholesterol (in relation to concentrations of carotenoids).

**2. Prospective associations of biomarker scores with incident T2D:** Country, centre, age, sex, BMI, waist circumference, smoking status, alcohol intake, education, physical activity, medical history (cancer, CHD, stroke), family history of diabetes, lipid-lowering medication, month of blood collection, dietary factors (total energy intake, other nutritional factors not included in calculations of respective dietary pattern indices will be considered), and in women, menopausal status and hormonal preparations use. We will also consider metabolic markers, i.e. markers of lipids (total cholesterol, HDL-cholesterol, LDL-cholesterol, triglycerides), inflammation (CRP), hepatic function (ALT, AST, GGT), and glycaemia (HbA1c, particularly to exclude those with raised HbA1c>48mmol/mol (or >6.5%). Final choice of covariates will be made with the goal of maximizing precision of the estimate for the main exposure without inducing a meaningful change in the estimate (>5% comparing models with and without a given covariate).

**Analysis Plan**

**Exclusions:** For the development of biomarker scores, we will restrict the analysis to the subcohort without missing data on nutritional biomarkers. Furthermore, EPIC-Oxford participants recruited via postal methods purposely oversampling vegetarians will be excluded in the primary analysis, and included as sensitivity analysis. Other exclusions will encompass: missing dietary data required for calculating dietary pattern indices; estimated daily energy intakes less than 800 kcal or more than 4,000 kcal for men and less than 500 kcal or more than 3500 kcal for women; prevalent cardiovascular disease or cancer, ferritin levels higher than 1,000 μg/L. Fatty acids with a mean concentration < 0.05% in the subcohort will not be used in the analyses.

For prospective associations of biomarker scores with T2D, participants with prevalent diabetes will be excluded.

**Descriptive statistics:** Descriptive statistics will be presented separately for each country. Measures of central tendency and dispersion or frequencies will be used, as appropriate, to describe sociodemographic and lifestyle characteristics, anthropometry, medical history, concentrations of (non-nutritional) metabolic markers, and concentrations of nutritional biomarkers included in the scores. These characteristics will also be presented within categories of adherence to dietary patterns, e.g. by quintiles.

**Biomarker scores derivation**: Food and nutrient intakes required for calculating dietary pattern scores will be estimated from food frequency questionnaires or diet histories (FFQ). Dietary pattern components with data unavailable in EPIC-InterAct will be excluded from score calculations (sodium for DASH and aHEI, trans-fatty acids for aHEI). Dietary pattern scores will be treated as continuous variables. Additionally, we will calibrate the intakes estimated from the FFQ against the 24h recalls (24-HR) administered in a random subset of the EPIC cohort using the following formula:

FFQ’ = 24-HR_mean_ + λ(FFQ – FFQ_mean_),

where 24-HR_mean_ and FFQ_mean_ denote population mean intake levels estimated by the two methods, and λ denotes the calibration factor. This factor represents the slope of the regression line obtained from linear regression of FFQ-estimated intake on the corresponding intake estimated from 24-HR [8,9]. Remaining analyses outlined below will be conducted both using uncalibrated and calibrated data, and the latter will be treated as secondary analyses as only a single 24-HR is available.

Biomarkers with highly right-skewed distributions will be log-transformed. Seasonality in 25(OH)D concentrations will be addressed by using a linear combination of sine and cosine functions of day of the year of blood draw. Carotenoids will be entered into the prediction models as residuals from linear regression models of individual carotenoids’ concentrations on total serum cholesterol concentrations. Concentrations of plasma fatty acids will be expressed as mole percentages.

Elastic net regression will be used to derive the biomarker scores in order to address collinearity between biomarkers and decrease data over-fitting in derivation datasets. Other regularization methods may also be considered. Because such methods account for linear combinations of predictors, we will evaluate potential non-linear associations between biomarkers and dietary patterns. In order to minimise the risk of data overfitting, we will use only restricted cubic splines with few knots. Similarly, we will undertake exploratory analyses to test all the possible first-order interaction terms simultaneously to verify the assumption of no interaction in a common prediction algorithm. Given the relative novelty of the topic of developing nutritional biomarker scores, we will also allow for other exploratory analyses or post-hoc analyses as indicated by results of the pre-specified analyses. If needed, such additional analyses will be agreed upon by the Working Group members and introduced as amendments to the statistical analysis plan prior to conducting them.

Considering goodness to fit and parsimony of a model, we will identify regression models to derive the biomarker scores, assign regression coefficients as weights to individuals’ data, and sum up the resulting products as a biomarker score. Performance of the equations will be assessed in the validation datasets by applying linear regression (non-penalized) and calculating R^2^. In order to assess whether personal characteristics identified in the literature as determinants of dietary misreporting (age, sex, BMI, and educational attainment) or factors influencing concentrations of some nutritional biomarkers (age, BMI, physical activity, smoking status, alcohol intake, medication use, season of blood draw) improve proportion of variance explained in adherence to dietary patterns, we will further include these factors in an additional model and calculate gains in R^2^. A backward deletion procedure at *P*-value = 0.10 will be applied to eliminate non-influential personal characteristics.

We will take at least two approaches to assess population-specific average internal validity and multi-national validity. For both types of validity, we will use an iterative procedure of developing the regression equations for biomarker scores in all but one study centre within country (internal validity) or country (multi-national validity) and testing their performance in the remaining study centre or country, respectively (validation dataset). However, the performance of the biomarker scores may vary among different population subgroups, and this approach may misrepresent performance of the biomarker scores. Thus, we will also assess validity by means of random splitting of the data. To aid interpretation of the results and potential differences between the two approaches, we will assess similarity between the study centres within countries, as well as between whole-country cohorts, with regards to dietary intakes, and sociodemographic and phenotypic characteristics of the participants.

Correlation coefficients between the biomarker scores and intakes of foods or nutrients used in calculations of dietary pattern adherence scores will be estimated.

**Prospective associations of the biomarker score with T2D:** Associations between the biomarker scores and incident T2D will be examined using multivariable adjusted, Prentice-weighted Cox regression. Country-specific estimates will be calculated, and pooled via random effects meta-analysis with stratification by discovery and validation datasets. We will assess departure from linearity using restricted cubic splines with the number of knots determined by the Akaike information criterion. As a complementary strategy to presenting Hazard Ratios, longitudinal results, T2D-free time from 50 years of age will be estimated across different degrees of the biomarker score.

We will fit the following models, with continuous covariates modelled using linear terms (in case of no evidence of departure from linearity) or restricted cubic splines (if non-linearity is detected or expected a priori, e.g. BMI):

Model 1 – adjusted for age, centre, sex, physical activity, smoking status, education level, total energy intake, alcohol.

Model 2 – as for model 1, plus BMI

Model 3 – as for model 2, plus dietary factors

Model 4 – as for model 3, plus metabolic factors

Analyses will be conducted using multiply-imputed, singly-imputed and non-imputed datasets (complete case-analysis) [10], depending on the degree and patterns of missing information. Multiple imputation will be applied to missing data in relation to covariates, as well as components of the biomarker score(s) at the stage of developing regression equations for calculating the score(s). Final values of the score(s) will not be directly imputed. We will undertake sensitivity analyses comparing the results between multiple imputation, single imputation, and complete-case analyses.

We will evaluate interactions by genetic risk scores for BMI, insulin resistance, T2D; age, sex and BMI. The list may be expanded with further identification of biologically plausible effect modifiers. Both multiplicative and additive interaction will be assessed.

Sensitivity analyses:

- Exclusion of participants with raised HbA1c>48mmol/mol (or >6.5%)
- Exclusion of T2D cases occurring in the first two years of follow-up
- Adjustment for foods or nutrients used for calculations of scores of adherence to dietary patterns

**Predictive modelling:** We will take at least two approaches to assess population-specific average internal validity and multi-national (internal-external) validity. Internal validity will be assessed by bootstrapping, rather than the split sample approach, in order to maximize statistical efficiency [11]. For the internal-external validation, we will use an iterative procedure of developing the prediction model in seven countries and validating it in the remaining country [12]. The so-called blow-up approach will be used to extrapolate case-cohort data to approximate T2D incidence.

A basic model will be specified with covariate selection based on established risk factors, i.e.: age, sex, smoking status, adiposity (BMI, waist circumference), physical activity, self-reported cardiometabolic disease, self-reported intakes of selected foods and metabolic markers (HbA1c, CRP, blood lipids, hepatic enzymes). Reproductive factors in females will be considered. The Royston-Sauerbrei’s D statistic will be used to estimate the proportion of variation explained by each model.

Utility of the biomarker score in improving risk prediction will be assessed by comparing gains in discrimination and calibration of models before and after inclusion of the score in the basic model. Use of individual biomarkers or reduced sets of biomarkers (to facilitate potential clinical application) will be considered upon developing the biomarker score(s).

Discrimination will be evaluated using the C-statistic and Net Reclassification Index, and calibration by plotting predicted survival curves against observed Kaplan-Meier curves in the validation dataset, as well as calculating the Brier score. Country-specific estimates and their heterogeneity will be summarised using random-effects meta-analysis. Additionally, subgroup analyses will be undertaken by sex, age (<60 years vs ≥60 years), BMI (<25 kg/m2 vs ≥25 kg/m2), and waist circumference (men <102 cm vs ≥102 cm; women <88 cm vs ≥88 cm).

**Cross-sectional analysis:** We will evaluate cross-sectional associations of the biomarker scores with circulating metabolic factors and anthropometry using linear-regression with adjustment for potential confounders (see Models 1 and 2 above). Given the moderate overlap in the nutritional biomarker panel between EPIC-InterAct and NHANES in years 2003-2006 and 2011-2014 [13], depending on the composition of the biomarker score(s) derived in the current study, corresponding cross-sectional analyses will be conceived among the Working Group members as a means of external validation [14].

**Amendments (summarised on 18/11/2022):**

1. *Use of raw data of individual fatty acids rather than publicly deposited data of groups of fatty acids*. Following gaining access to full biomarker data in the MedLey trial, EPIC-InterAct investigators reconsidered the “biomarker score of clinical intervention assignment” (Protocol 1) as our primary approach to derivation of a biomarker score of the Mediterranean diet, as reported in the current manuscript. Prior to this, we viewed the use of groupings of fatty acids in the MedLey trial (as available in the publicly deposited dataset) for external application of the biomarker score to EPIC-InterAct as too strong a threat to validity of the analysis to consider this type of biomarker score as our primary approach, because the composition of individual fatty acids within such groupings may have been fundamentally different between the two studies.

2. *Use of logistic elastic-net regression modelling individual biomarkers and their first-order interactions rather than use of standard logistic regression with backwards selection of individual biomarkers*. We increasingly recognised the utility of elastic net regression to develop a model predicting a dependent variable with multiple correlated predictors. We also found the assumption of no interactions between biomarkers in the score to be severely violated and we allowed for selection of predictors among all pairwise biomarker interactions in addition to log-linear terms. Because of the need to consider a pool of predictor variables exceeding the number of participants following this amendment, we applied logistic elastic net regression as our primary method of variable selection in lieu of logistic regression with backwards selection based on minimising the Bayesian information criterion.

3. *Method of variable selection stabilisation.* In the logistic elastic net regression analysis, we exchanged the bootstrap-enhanced stability selection of biomarkers for stability selection with repeated cross-validation. We selected the former a priori based on our earlier experience of observational derivation of biomarker scores using linear models (Protocol 2). It did not identify any biomarker terms consistently predictive of randomised assignment in the MedLey trial despite selection of multiple biomarker terms in individual runs of elastic net regression models. Upon additional reading of the literature, we found that bootstrapping for classification problems is suboptimal and favours overly parsimonious models [15]. This issue would be reasonably expected to be exacerbated by performing variable stability selection across bootstrap samples, which is consistent with our lack of identification of biomarker predictors using the bootstrap-based stability selection approach.

4. *Omission of secondary analyses*. We considered several secondary analyses as being of little benefit for our inference and our final report and omitted them: 1) computation of net re-classification index in the MedLey trial; 2) computation of relevant reclassification indices in the EPIC-InterAct; 3) assessment of utility of the biomarker score over and above established risk factors for prediction of type 2 diabetes; and 4) longitudinal analyses for incident outcomes in the EPIC-Norfolk study. 1) In the MedLey trial, C-statistic was considered as satisfactory to evaluate the performance of the derived biomarker scores. 2) In EPIC-InterAct, “validation” of the biomarker score was considered invalid to assess by using self-reported dietary indices as the reference measure because of the measurement error in subjective reporting and potentially limited construct validity of the Mediterranean diet score. 3) To make the manuscript succinct and focused on the single research aim, we decided not to explore whether inclusion of the nutritional biomarkers would merit prediction of type 2 diabetes risk currently available in a clinical setting. Lastly, 4) the EPIC-Norfolk study is a part of EPIC-InterAct, ascertaining the incidence of a number of disease endpoints, not only type 2 diabetes (n diabetes cases = 681). For the purpose of simplicity and clear focus on type 2 diabetes as the primary outcome in the EPIC-InterAct (n cases = 9,453 in the current study), we decided to reserve any longitudinal analyses for multiple outcomes in the EPIC-Norfolk Study for future publications.

**Additional amendment (08/02/23):**

Because of a request in the peer-review process, we have now performed the analysis of discriminatory performance of the biomarker score for high versus low self-reported adherence to the Mediterranean diet. This analysis was previously omitted as described under point four of the above section “Amendments (summarised on 18/11/2022).”

**References**

1. Davis C, Hodgson J, Bryan J, Garg M, Woodman R, Murphy K. Older Australians Can Achieve High Adherence to the Mediterranean Diet during a 6 Month Randomised Intervention; Results from the Medley Study. Nutrients. 2017;9: 534. doi:10.3390/nu9060534

2. Langenberg C, Sharp S, Forouhi NG, Franks PW, Schulze MB, Kerrison N, et al. Design and cohort description of the InterAct Project: An examination of the interaction of genetic and lifestyle factors on the incidence of type 2 diabetes in the EPIC Study. Diabetologia. 2011;54: 2272–2282. doi:10.1007/s00125-011-2182-9

3. Day N, Oakes S, Luben R, Khaw K-T, Bingham S, Welch A, et al. EPIC-Norfolk: Study design and characteristics of the cohort. Br J Cancer. 1999;80 Suppl 1: 95–103. doi:10466767

4. Song X, Huang Y, Neuhouser ML, Tinker LF, Vitolins MZ, Prentice RL, et al. Dietary long-chain fatty acids and carbohydrate biomarker evaluation in a controlled feeding study in participants from the Women’s Health Initiative cohort. Am J Clin Nutr. 2017;105: 1272–1282. doi:10.3945/ajcn.117.153072

5. Cho S, Kim K, Kim YJ, Lee JK, Cho YS, Lee JY, et al. Joint Identification of Multiple Genetic Variants via Elastic-Net Variable Selection in a Genome-Wide Association Analysis. Ann Hum Genet. 2010;74: 416–428. doi:10.1111/j.1469-1809.2010.00597.x

6. Zheng J-S, Imamura F, Sharp SJ, van der Schouw YT, Sluijs I, Gundersen TE, et al. Association of plasma vitamin D metabolites with incident type 2 diabetes: EPIC-InterAct case-cohort study. J Clin Endocrinol Metab. 2018 [cited 14 Nov 2018]. doi:10.1210/jc.2018-01522

7. Forouhi NG, Koulman A, Sharp SJ, Imamura F, Kröger J, Schulze MB, et al. Differences in the prospective association between individual plasma phospholipid saturated fatty acids and incident type 2 diabetes: The EPIC-InterAct case-cohort study. Lancet Diabetes Endocrinol. 2014;2: 810–818. doi:10.1016/S2213-8587(14)70146-9

8. Kaaks R, Riboli E, van Staveren W. Calibration of dietary intake measurements in prospective cohort studies. Am J Epidemiol. 1995;142: 548–56. Available: http://www.ncbi.nlm.nih.gov/pubmed/7677134

9. Rosner B, Spiegelman D, Willett WC. Correction of logistic regression relative risk estimates and confidence intervals for random within-person measurement error. Am J Epidemiol. 1992;136: 1400–1413. doi:10.1093/oxfordjournals.aje.a116453

10. Sterne JAC, White IR, Carlin JB, Spratt M, Royston P, Kenward MG, et al. Multiple imputation for missing data in epidemiological and clinical research: Potential and pitfalls. BMJ. 2009;339: 157–160. doi:10.1136/bmj.b2393

11. Steyerberg EW, Harrell FE. Prediction models need appropriate internal, internal-external, and external validation. J Clin Epidemiol. 2016;69: 245–247. doi:10.1016/j.jclinepi.2015.04.005

12. Royston P, Parmar MKB, Sylvester R. Construction and validation of a prognostic model across several studies, with an application in superficial bladder cancer. Stat Med. 2004;23: 907–926. doi:10.1002/sim.1691

13. Pfeiffer CM, Lacher DA, Schleicher RL, Johnson CL, Yetley EA. Challenges and Lessons Learned in Generating and Interpreting NHANES Nutritional Biomarker Data. Adv Nutr An Int Rev J. 2017;8: 290–307. doi:10.3945/an.116.014076

14. Imamura F, Sharp SJ, Koulman A, Schulze MB, Kröger J, Griffin JL, et al. A combination of plasma phospholipid fatty acids and its association with incidence of type 2 diabetes: The EPIC-InterAct case-cohort study. PLoS Med. 2017;14: 1–19. doi:10.1371/journal.pmed.1002409

15. Ounpraseuth S, Lensing SY, Spencer HJ, Kodell RL. Estimating misclassification error: A closer look at cross-validation based methods. BMC Res Notes. 2012;5. doi:10.1186/1756-0500-5-656
